# The Neuroprotective Effect of 4-Octyl Itaconate on Acute Period of Experimental Autoimmune Neuritis

**DOI:** 10.1007/s10753-025-02370-w

**Published:** 2025-12-17

**Authors:** Lin-Jie Zhang, Ning Zhao, Jia Li, Hui Zhai, Jie Wu, Li Yang

**Affiliations:** 1 Department of Neurology, Tianjin Neurological Institute, Tianjin Medical University General Hospital, Tianjin, 300052 China; 2 Department of Neurology, Zhuhai People’s Hospital, Zhuhai, 519000 China

**Keywords:** Guillain-Barré syndrome, Experimental autoimmune neuritis, Macrophages, 4-Octyl itaconate, NLRP3 inflammasome

## Abstract

The current standard therapy for acute-phase Guillain-Barré syndrome (GBS) remains limited to intravenous immunoglobulin and plasma exchange. Developing novel pharmacological agents to halt acute disease progression represents a critical unmet need. 4-Octyl itaconate (4-OI), a cell-permeable itaconate derivative, has emerged as a promising compound with anti-inflammatory and antioxidant properties. This study aimed to investigate the therapeutic effects and mechanisms of 4-OI in the acute experimental autoimmune neuritis (EAN) model. Throughout the EAN disease course, clinical and electrophysiological assessments were conducted. Inflammatory infiltration and demyelination in sciatic nerves were evaluated by Hematoxylin-Eosin staining and Luxol Fast Blue staining, respectively. The inflammatory response and macrophage polarization were analyzed by quantitative polymerase chain reaction, western blot, and immunofluorescence. Bone marrow-derived macrophages (BMDMs) were used to elucidate the immunomodulatory mechanisms of 4-OI in vitro. 4-OI treatment significantly improved clinical scores and attenuated neuropathological damage in EAN rats. It reduced inflammatory infiltration, M1 macrophage polarization, NLRP3 inflammasome activation, and oxidative stress in the peripheral nerves. In BMDMs, 4-OI inhibited LPS/IFN-γ-induced M1 polarization, inflammatory cytokine release, and oxidative stress. These effects were mediated by activation of the Nrf2/HO-1 pathway, as the Nrf2 inhibitor ML385 partially reversed the protective effects of 4-OI. 4-OI alleviates acute-phase EAN through its anti-inflammatory and antioxidant activities, which are largely dependent on the Nrf2/HO-1 pathway. These preclinical findings highlight 4-OI as a promising candidate for the treatment of GBS, supporting its further translational development.

**Supplementary Information:**

The online version contains supplementary material available at 10.1007/s10753-025-02370-w.

## Introduction

Guillain-Barré syndrome (GBS) is an acute immune-mediated peripheral neuropathy that typically peaks in severity within two weeks. The disease carries substantial risks, with 20%−30% of patients requiring mechanical ventilation and approximately 5% facing mortality. Although 60–80% of patients regain independent walking within six months of onset [1–3], treatment options remain limited. Current standard care relies on plasma exchange and intravenous immunoglobulin (IVIG), yet neither extended nor repeated courses of these interventions have shown significant additional benefit in severe cases [4]. Emerging approaches such as FcRn antagonists are under investigation, though their clinical efficacy remains unestablished. These constraints highlight the urgent need for novel therapeutic strategies to improve outcomes in GBS.

The pathological manifestations of GBS encompass a spectrum of neuroinflammatory changes, including neuroedema, perivenous lymphocyte infiltration, macrophage-mediated demyelination, and deposition of antibodies and membrane attack complexes [5]. Central to this process are infiltrating macrophages, which play a pivotal and multifaceted role in nerve damage. They mediate myelin sheath destruction and secrete pro-inflammatory chemokines and cytokines [6]. This inflammatory milieu is further amplified by complement system activation triggered by autoantibodies [7] and by T cells through TNF-α secretion, which upregulates adhesion molecules and chemokines to facilitate further macrophage recruitment [8, 9]. Together, these events establish a self-perpetuating cycle of inflammatory amplification and demyelination. The activity of these macrophages exhibits functional polarization, with the pro-inflammatory M1 phenotype being particularly implicated. M1 macrophages express inflammatory mediators and generate reactive oxygen and nitrogen species in response to local damage signals [6]. Histopathological evidence from sural nerve biopsies of acute inflammatory demyelinating polyradiculoneuropathy (AIDP) patients consistently reveals macrophage-associated demyelinating lesions, often initiating at sites of complement deposition [10]. These findings collectively underscore the central role of macrophage-driven inflammation in the pathogenesis and progression of GBS.

Itaconate is a key metabolite in classically activated macrophages, acting not only as a metabolic intermediate but also as a potent regulator of pro-inflammatory function [11]. Given the therapeutic implications of this regulatory role, the cell-permeable derivative 4-octyl itaconate (4-OI) serves as a valuable experimental tool due to its functional similarity to endogenous itaconate. Therefore, evaluating the therapeutic potential of 4-OI in GBS through macrophage modulation represents a promising strategy, yet its efficacy and underlying mechanisms require thorough investigation.

## Materials and Methods

### Animal Studies

Experimental Autoimmune Neuritis (EAN) rat is a widely used animal model of AIDP subtype in GBS [12]. Lewis rats (6–8 weeks, 160–180 g; Beijing Vital River Laboratory Animal Technology Co., Ltd) were immunized in the hind footpads with a 300 µL emulsion containing 300 µg P0_180 − 199_ peptide (GenScript, China) and 2.4 mg M. tuberculosis H37RA (BD, USA, cat#231141) in complete Freund’s adjuvant (Sigma, USA, cat#F5881). The rats were randomly assigned to three groups (*n* = 6): CON (normal saline), EAN (normal saline), and EAN + 4-OI (100 mg/kg 4-OI, MCE, cat#HY-112675). The dose we selected was based on published animal studies and was further refined after preliminary experiments [13]. From day 3 to 16 post-immunization (P3–P16), the EAN + 4-OI group received daily intraperitoneal injections of 4-OI. A separate researcher, who was not involved in any assessments, randomized the animals, coded them with unique numbers, and administered all treatments. Body weight and neurological scores (0–10 scale) were recorded daily by two experimenters who were blinded to the group allocation of the animals. On P16, all animals underwent neurophysiological examination prior to collection of serum and sciatic nerve samples.

### Cell Experiments

Bone marrow-derived macrophages (BMDMs) from Lewis rats [14] were cultured in DMEM (Gibco, China, cat#C11965500BT) supplemented with 10% FBS (Pricella, China, cat#164250) and 20 ng/mL M-CSF (PeproTech, USA, cat#400-28). The dose of 4-OI (125 µM and 250 µM) were determined by CCK-8 assay and relevant reference [15]. To induce M1 polarization, cells were stimulated with LPS (200 ng/ml, Solarbio, China, cat#L8880) and IFN-γ (50 ng/ml, PeproTech, USA, cat#AF-315-05). 4-OI (125 or 250 µM) or the Nrf2 inhibitor ML385 (5 µM, AbMole, USA, cat#M8692) was added 1 h prior to LPS/IFN-γ stimulation. The experimental design included control, 4-OI (250 µM), model (LPS/IFN-γ), intervention (125 or 250 µM 4-OI + LPS/IFN-γ) groups. In a separate set of experiments to verify Nrf2 dependence, control, ML385 (5 µM) [16], model, intervention (250 µM 4-OI + LPS/IFN-γ), inhibition (250 µM 4-OI + ML385 + LPS/IFN-γ) groups were established. After treatment, supernatants and cells were collected for analysis. All results are representative of three independent biological replicates (*n* = 3).

### Harvest of BMDMs

Lewis rats, 6- to 8-week-old, were euthanized, and the femurs and tibiae were aseptically dissected. The bone marrow cavity was flushed thoroughly with cold, sterile PBS using a sterile syringe. The flushed suspension was passed through a 70-µm cell strainer. The bone marrow progenitor cells were harvested and cultured in complete medium supplemented with 20 ng/mL M-CSF. On day 3, half of the culture medium was replaced with fresh medium. A complete medium change was performed on day 5. The cells matured and differentiated into BMDMs by day 7.

### Neurological Function Scores and Electrophysiological Examination

Neurological function was assessed daily using a 10-point scale: 0, normal; 1, reduced tail reflexes; 2, tail paralysis/impaired righting; 3, absent righting; 4, ataxic gait/abnormal paw position; 5, mild hindlimb paralysis; 6, moderate hindlimb paralysis; 7, severe hindlimb paralysis; 8, mild tetraparesis; 9, severe tetraparesis or moribund; 10, death.

On P16, rats under anesthesia underwent neurophysiological examination with a fully digital KeyPoint Compact EMG/NCS/EP recording system (Dantec). After sciatic nerve exposure, compound muscle action potentials (CMAP) amplitude, motor nerve conduction velocity (MNCV), and distal latency (DL) were recorded.

### Cell Counting Kit-8 (CCK-8) Assay

BMDMs (1 × 10³/well) were treated with 4-OI (31.25–500 µM) for 24 h, then incubated with CCK-8 reagent (4 h). Absorbance (450 nm) was measured on the microplate reader (Thermo Fisher, USA).

### Histopathological Analysis

Sciatic nerves were sectioned (6 μm) and stained using the Hematoxylin-Eosin staining (HE) and Luxol fast blue (LFB) kits (Solarbio, Beijing). HE was for inflammatory cells infiltration and LFB for demyelination. Severity was graded as follow: 0, almost no infiltration of inflammatory cells or loss of myelin sheath; 1, a few scattered inflammatory cells or mild demyelination; 2, aggregation of inflammatory cells and moisture accumulation around blood vessels or moderate demyelination; and 3, extensive infiltration of inflammatory cells or severe demyelination.

### Immunofluorescence Staining

Tissue frozen sections and cell climbing sheets were stained by primary antibodies (MBP, 1:500, Proteintech, China,

cat#10458-1-AP; CD68, 1:200, Santa Cruz, USA, cat#sc-20060; CD86, 1:200, Abclonal, China, cat#A21198; iNOS, 1:200, Abcam, USA, cat#ab178945), fluorescent secondary antibodies, and DAPI (Abcam, USA, cat#ab285390), then observed by fluorescence microscopy (Olympus, Japan).

### MDA, SOD, ROS Detection

MDA, SOD and ROS levels were quantified using commercial kits (Beyotime, China).

### Quantitative Real-Time Polymerase Chain Reaction (qPCR) and Western Blotting (WB)

The RNA were extracted using TRIzol (Invitrogen, USA, cat#15596018), cDNA was prepared using reverse-transcription kits (Vazyme, China), and amplified using 2×RealStar Fast SYBR qPCR Mix (Genstar, China) on QuantStudio 3 RT-PCR System (Thermo Fisher, USA). Data were calculated using the 2^−ΔΔCt^ method.

Tissue proteins were separated using TRIzol, and cell proteins using RIPA buffer (Solarbio, China,). Proteins were separated by SDS-PAGE, transferred to PVDF membranes (Millipore, USA), and probed with primary antibodies against β-actin (1:1000, CST, USA, cat#4967), Nrf2 and HO-1 (1:1000, Proteintech, China, cat#16396-1-AP, 10701-1-AP), NLRP3 and caspase-1 (1:1000, CST, USA, cat#15101, 83383), ASC (1:800, Abclonal, China, cat#A24165), iNOS (1:1000, Abcam, USA, cat#178945), then secondary antibody (horseradish peroxidase-conjugated goat anti-rabbit, 1:5000). Finally, the gel imaging system (Bio-Rad) and an enhanced chemiluminescence kit (Millipore, USA) were used for developing the protein bands. The ImageJ software was used to measure the grayscale values.

### Enzyme-Linked Immunosorbent Assay (ELISA)

The levels of IL-1β (Proteintech, China, cat#KE20021) and IL-18 (ELK, China, cat#ELK2270) in serum and supernatants were measured according to the manufacturers’ protocols.

## Statistical Analysis

The data were analyzed and graphs were constructed using GraphPad Prism software (V9.1.1). Unpaired T-test, Welch T-test, or Mann Whitney U-test was used to compare two independent samples. One-way ANOVA or Kruskal Wallis H-test was used for multiple group comparisons, and Dunnett or Tukey methods was used for multiple comparison P-value correction. Data are expressed as the mean ± SEM. *P* < 0.05 represented as statistically significant differences.

## Results

### In Vivo Experiments

#### 4-OI Alleviated Acute Phase of EAN Rats

**Fig. 1:**
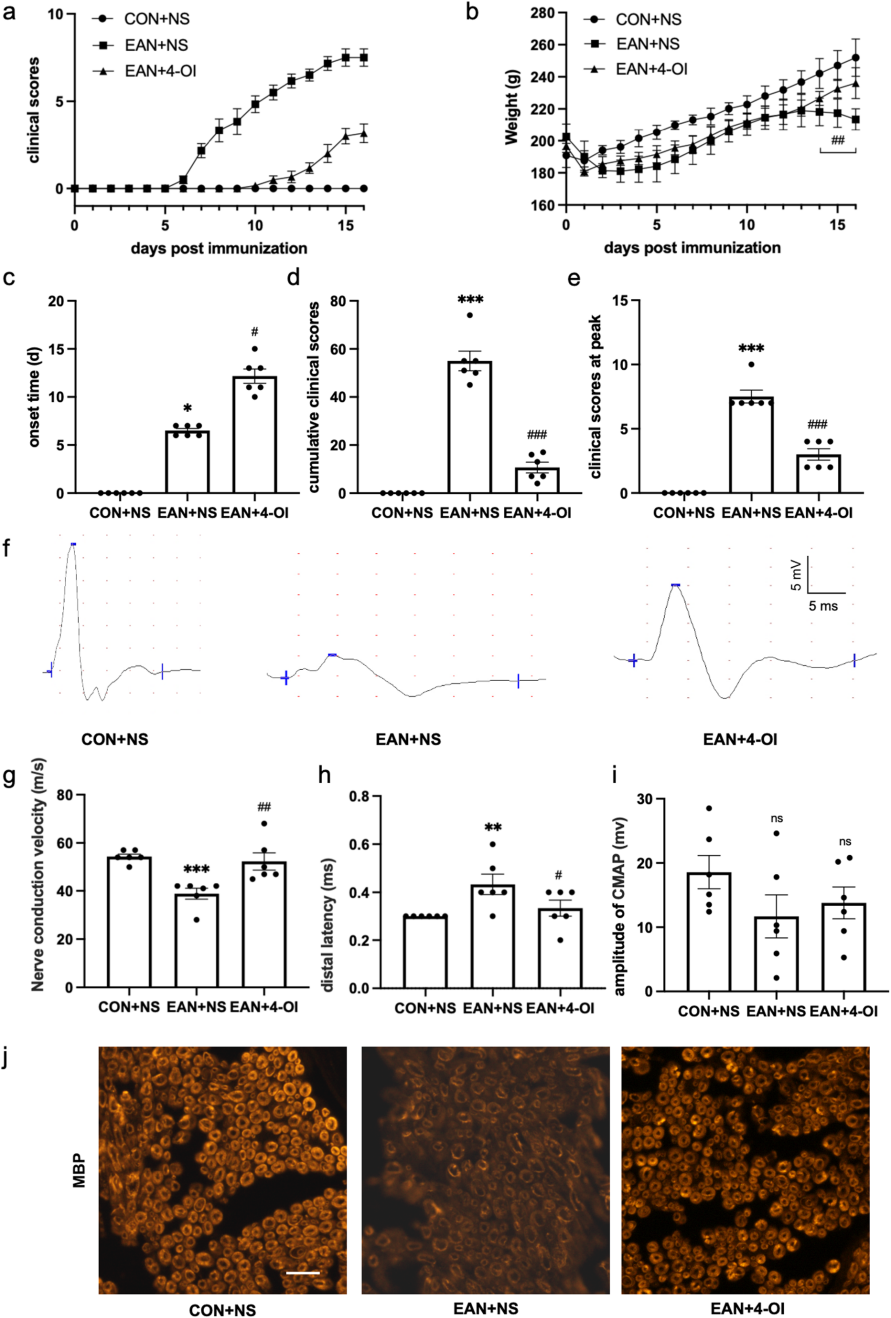
4-OI alleviated acute-phase EAN. (**a**)-(**e**): 4-OI delayed the onset time (*P* < 0.05), reduced the neurological scores (*P* < 0.001, *P* < 0.001) of EAN. (**b**): 4-OI alleviated the weight loss of EAN (*P* < 0.01). (**f**)-(**i**): 4-OI improved the nerve conduction velocity (*P* < 0.01), distal latency (*P* < 0.05), CMAP amplitude of EAN (*P* > 0.05). (**j**): The expression of MBP reduced in EAN, and 4-OI increased the expression. Scale bar = 20 μm, *n* = 6. EAN, experimental autoimmune neuritis; CMAP, compound muscle action potential; NS: normal saline. *P < 0.05, **P < 0.01, ***P < 0.001, EAN + NS vs. CON + NS. ^#^ *P *< 0.05, ^##^ *P *< 0.01, ^###^ *P *< 0.001, EAN + NS vs. EAN + 4-OI. ns, *P* > 0.05

From P0 to P16 day, rats in the CON group remained asymptomatic, whereas both the EAN and EAN + 4-OI groups began to develop neurological symptoms between P6 and P11 (Fig. 1a). Body weight increased steadily in the CON group, but decreased initially before recovering in the EAN and EAN + 4-OI groups. Notably, during the peak disease period, the EAN + 4-OI group maintained a significantly higher body weight than the EAN group (*P* < 0.01) (Fig. 1b). The onset of disease in the EAN + 4-OI group was significantly delayed compared to the EAN group (12.17 ± 0.75 vs. 6.5 ± 0.22 days, *P* < 0.05,Fig. 1c). Furthermore, the EAN + 4-OI group showed markedly lower neurological impairment than the EAN group, as reflected in both the cumulative score (10.67 ± 2.20 vs. 55.00 ± 4.09, *P* < 0.001, Fig. 1d) and the peak score (3.00 ± 0.45 vs. 7.50 ± 0.50, *P* < 0.001, Fig. 1e).

#### 4-OI Alleviated Sciatic Nerve Injury in EAN Rats

Neurophysiological assessment of sciatic nerve function revealed that EAN rats exhibited significantly slower NCV, reduced CMAP amplitude, and prolonged DL compared to the CON group (Fig. 1f-i). Specifically, the EAN group showed markedly slower NCV (38.83 ± 2.26 m/s) than the CON group (54.33 ± 1.05 m/s, *P* < 0.001), which was significantly improved by 4-OI treatment (52.33 ± 3.58 m/s, *P* < 0.01). Similarly, DL was longer in the EAN group (0.43 ± 0.04 ms) than the CON group (0.30 ± 0.00 ms, *P* < 0.01), and shortened in the EAN + 4-OI group (0.33 ± 0.03 ms, *P* < 0.05). Although CMAP amplitude was lower in the EAN group (11.68 ± 3.35 mV) than in the CON (18.57 ± 2.59 mV) and EAN + 4-OI (13.78 ± 2.48 mV) groups, the difference was not statistically significant (*P* > 0.05), suggesting the absence of severe axonal injury. Consistent with the functional deficits, MBP levels in sciatic nerves were significantly reduced in the EAN group compared to both the CON and the EAN + 4OI groups (Fig. 1j), indicating demyelination that was ameliorated by 4-OI treatment.

####  4-OI Reduced Inflammatory Cell Infiltration and Demyelination in the Sciatic Nerve of EAN Rats

**Fig. 2:**
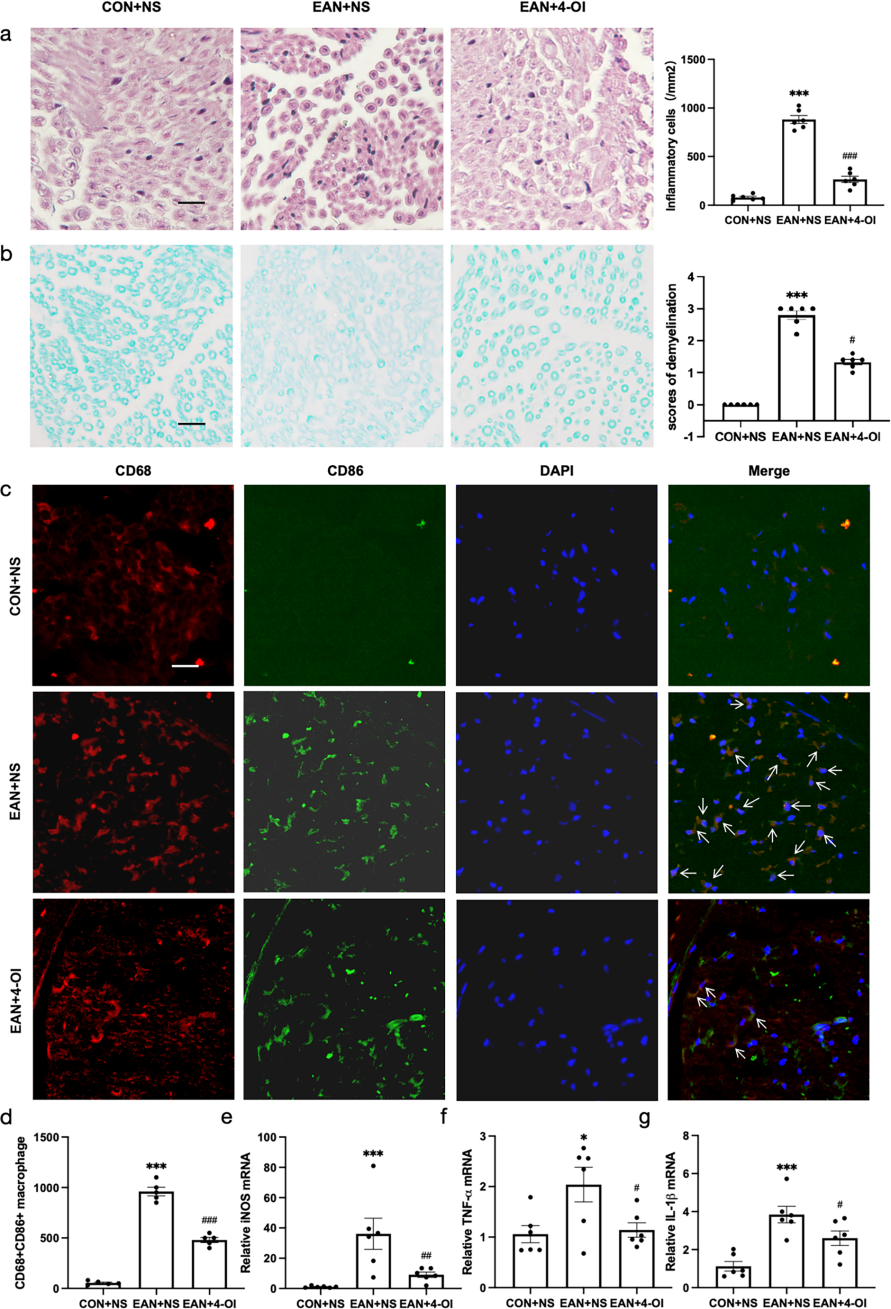
4-OI suppressed the inflammatory infiltration and demyelination of EAN and reduced the M1 macrophage. (**a**) HE showed that 4-OI reduced the infiltration of inflammatory cells (*P* < 0.001). (**b**): LFB demonstrated that 4-OI alleviated the degree of demyelination (*P *< 0.05). (**c**)-(**d**): 4-OI reduced the infiltration of M1 macrophage in the sciatic nerve of EAN (*P* < 0.001). (**e**)-(**g**): 4-OI inhibited the mRNA expression of the markers of M1 macrophage (iNOS, TNF-α, and IL-1β) in spleen mononuclear cells of EAN (*P* < 0.01, *P* < 0.05, *P* < 0.05). Scale bar = 20 μm, *n* = 6. EAN, experimental autoimmune neuritis; HE, Hematoxylin-Eosin staining; LFB, Luxol fast blue. NS: normal saline. *P < 0.05, **P < 0.01, ***P < 0.001, EAN + NS vs. CON + NS. ^#^ *P *< 0.05, ^##^ *P *< 0.01, ^###^ *P *< 0.001, EAN + NS vs. EAN + 4-OI

Histopathological analysis of sciatic nerves revealed significantly more inflammatory cell infiltration in the EAN group than in the CON and EAN + 4-OI groups (*P* < 0.001,Fig. 2a). Consistent with this, LFB staining showed severe demyelination in the EAN group (2.80 ± 0.14), which was significantly attenuated in the EAN + 4-OI group (1.33 ± 0.08, *P* < 0.05). No demyelination was observed in the CON group (Fig. 2b).

#### 4-OI Reduced M1 Macrophages in the Sciatic Nerve and Spleen of EAN Rats

Immunofluorescence staining revealed a marked increase in M1 macrophages (CD68 + CD86+) in the sciatic nerves of the EAN group compared to the CON group (*P* < 0.001), which was significantly suppressed in the EAN + 4-OI group (*P* < 0.001 Fig. 2c-d). Consistently, the mRNA expression of M1-associated markers (iNOS, TNF-α, and IL-1β) in splenic mononuclear cells was significantly up-regulated in the EAN group (*P* < 0.05) and down-regulated in the EAN + 4-OI group (*P* < 0.05, Fig. 2e-g).

#### 4-OI Inhibited Inflammatory Factors in the Sciatic Nerve and Serum of EAN Rats

**Fig. 3:**
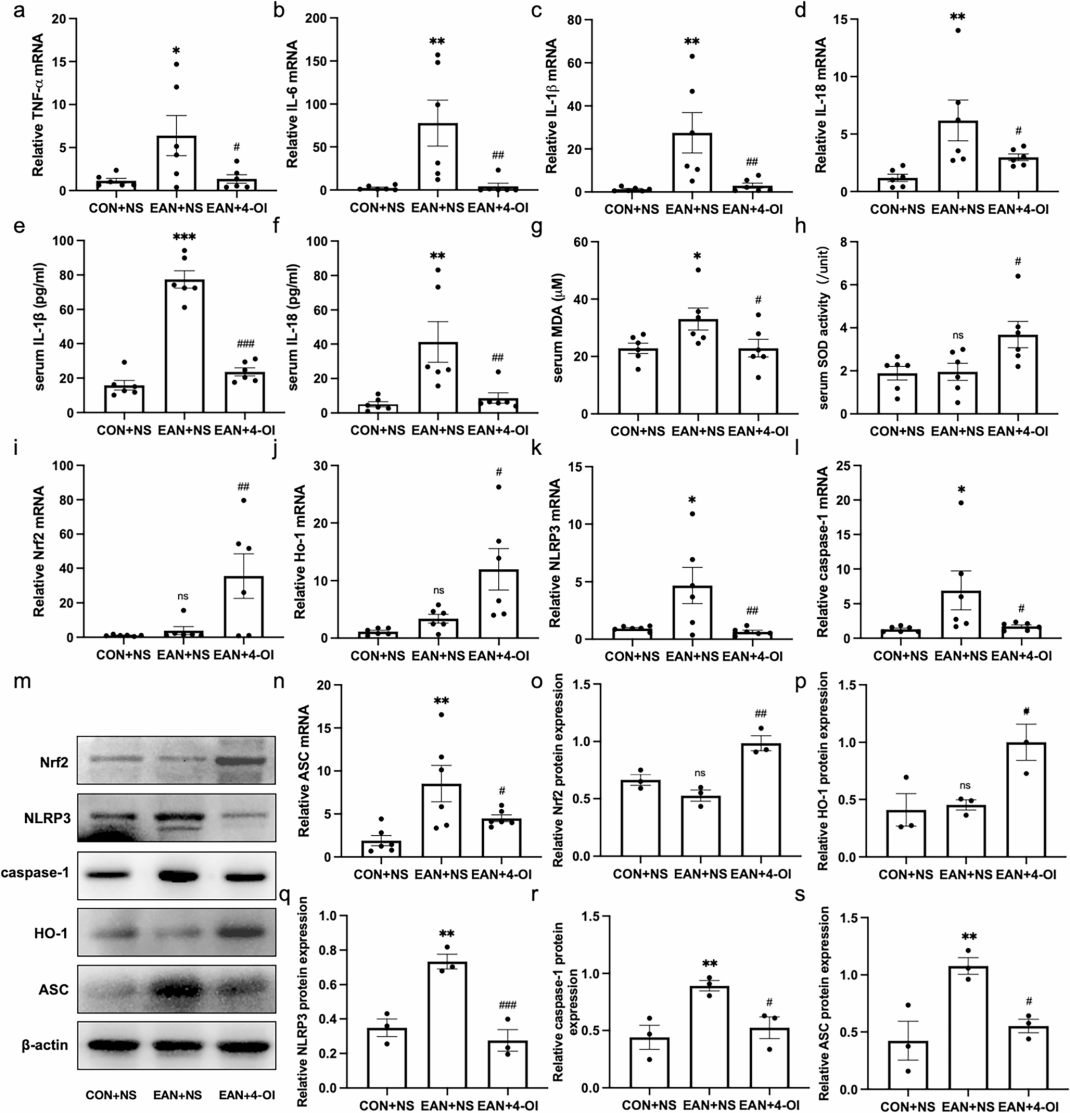
4-OI reduced inflammatory factors, oxidative stress and NLRP3 inflammasome activation in EAN. (**a**)-(**d**): 4-OI inhibited the mRNA expression of TNF-α (*P* < 0.05), IL-6 (*P* < 0.01), IL-1β (*P* < 0.01), and IL-18 (*P* < 0.05) in the sciatic nerve of EAN. (**e**)-(**f**): 4-OI reduced the serum levels of IL-1β (*P* < 0.001) and IL-18 (*P* < 0.01). (**g**)-(**h**): 4-OI reduced the serum MDA levels (*P* < 0.05) and increased the SOD activity (*P* < 0.05). (**i**)-(**s**): The mRNA and protein expression levels of Nrf2/HO-1, NLRP3, caspase-1, and ASC in the sciatic nerve of each group. 4-OI increased the expression of Nrf2/HO-1 (*P* < 0.05), and inhibited the expression of NLRP3, caspase-1, and ASC (*P* < 0.05). *n* = 6. EAN, experimental autoimmune neuritis. NS: normal saline. *P < 0.05, **P < 0.01, ***P < 0.001, EAN + NS vs. CON + NS. ^#^ *P *< 0.05, ^##^ *P *< 0.01, ^###^ *P *< 0.001, EAN + NS vs. EAN + 4-OI

The mRNA levels of inflammatory cytokines (TNF-α, IL-6, IL-1β, and IL-18) in sciatic nerves were significantly elevated in the EAN group compared to the CON group (*P* < 0.05) but were markedly reduced in the EAN + 4-OI group (*P* < 0.05, Fig. 3a-d). Consistent with these findings, serum levels of IL-1β and IL-18 were also significantly higher in the EAN group (*P* < 0.01) and significantly decreased following 4-OI administration (*P* < 0.01, Fig. 3e-f).

#### 4-OI Inhibited Oxidative Stress in EAN Rats

4-OI treatment significantly ameliorated oxidative stress in the EAN + 4-OI group, as evidenced by a reduction in serum MDA levels (*P* < 0.05, Fig. 3g) and an increase in serum SOD activity (*P* < 0.05, Fig. 3h) compared to the EAN group. While MDA was significantly elevated in the EAN group relative to the CON group (*P* < 0.05), SOD activity remained unchanged between these two groups.

#### 4-OI Inhibited the NLRP3 Inflammasome and Activated the Nrf2/HO-1 Signaling Pathway

WB and qPCR analyses revealed that 4-OI treatment significantly up-regulated the Nrf2/HO-1 pathway and suppressed the NLRP3 inflammasome in sciatic nerves (Fig. 3i-s). Compared with the EAN group, the EAN + 4-OI group showed increased mRNA and protein expression of Nrf2 and HO-1, while the expression of NLRP3, caspase-1, and ASC was significantly decreased (*P* < 0.05). No significant differences in Nrf2/HO-1 levels were observed between the EAN and CON groups.

### In Vitro Experiments

#### 4-OI Inhibited M1 Polarization of BMDMs

**Fig. 4:**
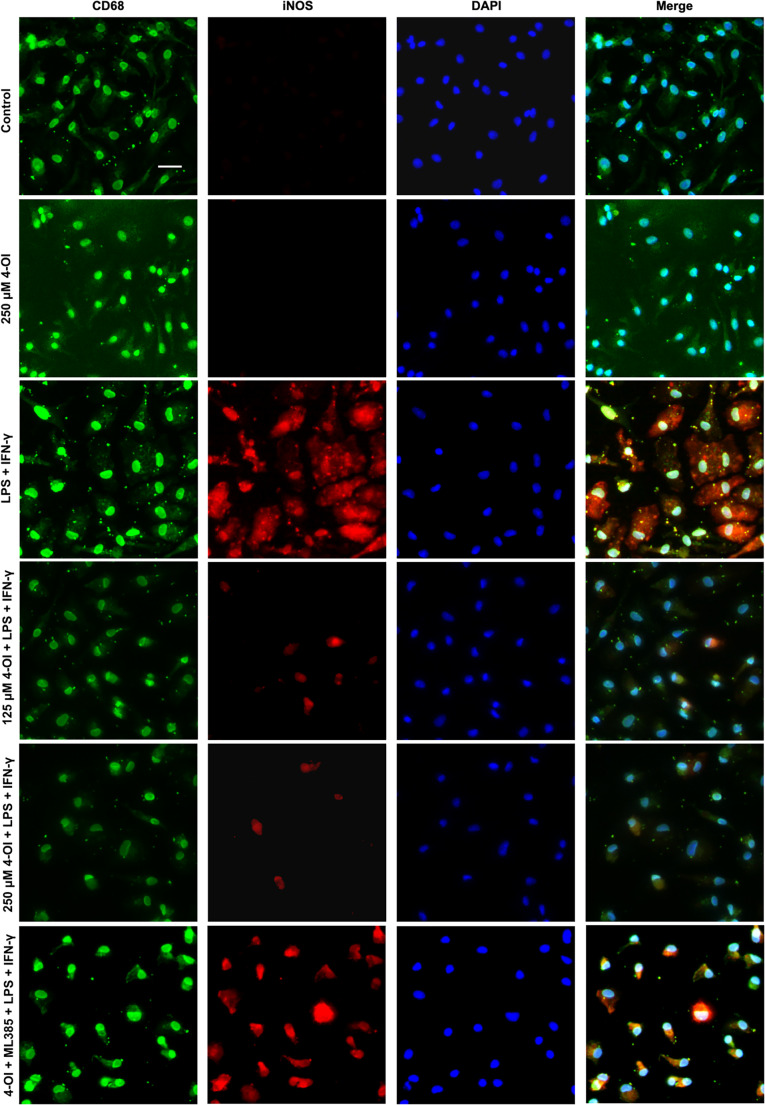
4-OI inhibited M1 polarization of BMDM and be partially reversed by ML385. 4-OI inhibited the number of CD68 + iNOS + BMDM and ML385 increased the number of CD68 + iNOS + BMDM. Scale bar = 20 μm, *n* = 3. BMDM, bone marrow derived macrophage

**Fig. 5:**
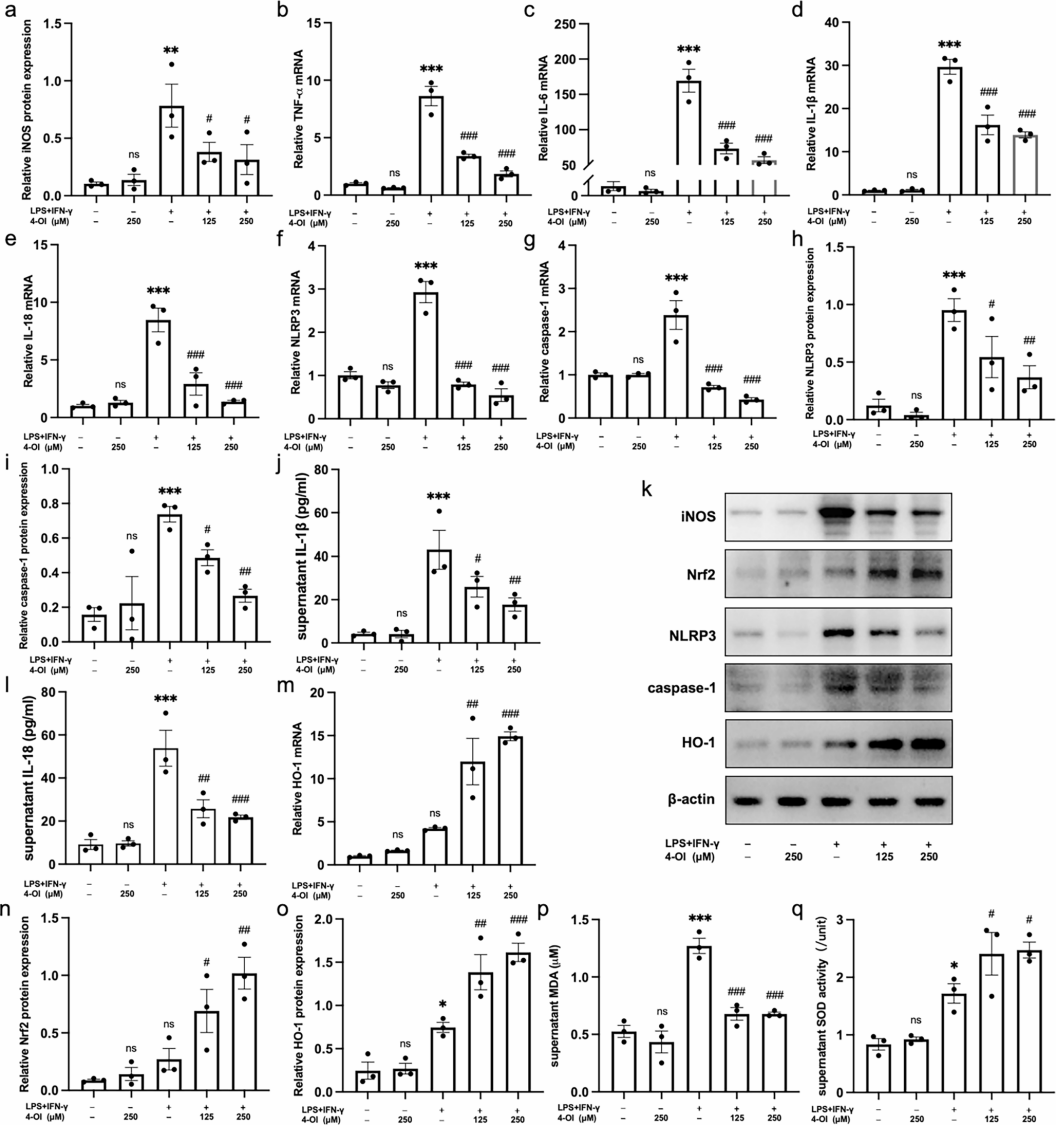
4-OI inhibited the M1 polarization and oxidative stress of BMDM. (**a**, **k**): 4-OI inhibited the protein expression of iNOS (*P* < 0.05). (**b**)-(**e**): 4-OI inhibited the mRNA expression of inflammatory factors (TNF-α, IL-6, IL-1β, and IL-18) (*P* < 0.001). (**f**)-(**i**)(**k**): 4-OI inhibited the mRNA and protein expression of NLRP3 and caspase-1 in BMDM (*P* < 0.05). (**j**)-(**l**): 4-OI inhibited the secretary levels of IL-1β and IL-18 in the cultural supernatants (*P* < 0.05). (**m**)-(**o**)(**k**): 4-OI activated the Nrf2/HO-1 signaling pathway (*P* < 0.05). (**p**)-(**q**): 4-OI inhibited the MDA levels (*P* < 0.001) and increased the SOD activity (*P* < 0.05) of BMDM. *n* = 3. BMDM, bone marrow derived macrophage. *P < 0.05, **P < 0.01, ***P < 0.001, the model group vs. the control group. ^#^ *P *< 0.05, ^##^ *P *< 0.01, ^###^ *P *< 0.001, the model group vs. the intervention groups. ns, *P* > 0.05, the 4-OI group vs. the contol group

Immunofluorescence staining of CD68 and iNOS showed that the number of M1 macrophages was substantially increased in the model group but markedly reduced by 4-OI treatment (Fig. 4). Consistent with this, WB analysis confirmed that the protein expression of iNOS, a specific M1 marker, was significantly up-regulated in the model group compared to the control group (*P* < 0.01), and significantly down-regulated in the intervention groups (*P* < 0.05, Fig. 5a, k).

#### 4-OI Inhibited Inflammatory Factors Production and NLRP3 Inflammasome Activation in BMDMs

4-OI treatment significantly suppressed the LPS/IFN-γ-induced inflammatory response in BMDMs. The mRNA levels of TNF-α, IL-6, IL-1β, and IL-18, along with the mRNA and protein expression levels of NLRP3 and caspase-1 were markedly elevated in the model group (*P* < 0.001) and significantly reduced by 4-OI.(*P* < 0.05, Fig. 5b-i, k). Consistent with the inhibition of the NLRP3 inflammasome pathway, the secretion of its downstream products, IL-1β and IL-18, in the supernatants were also significantly reduced in the intervention groups (*P* < 0.05, Fig. 5j, l).

#### 4-OI Inhibited Oxidative Stress in BMDMs

**Fig. 6:**
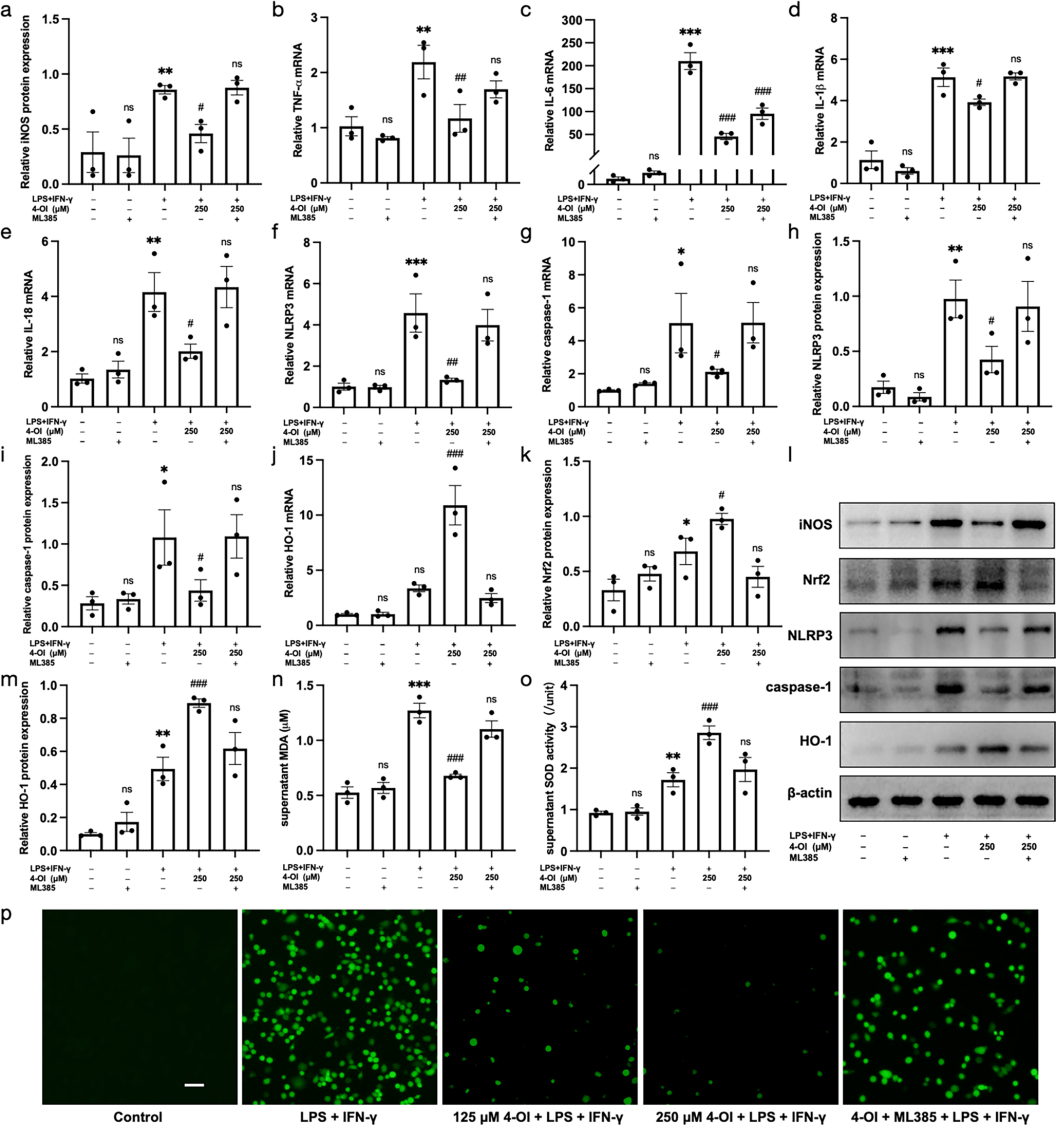
4-OI inhibited inflammation and oxidative stress largely via Nrf2/HO-1 pathway. (**a**, **l**): ML385 treatment partially reversed the inhibitory effect of 4-OI on the protein expression of iNOS (*P* < 0.05). (**b**)-(**e**): ML385 treatment partially reversed the inhibitory effect of 4-OI on the mRNA levels of inflammatory factors (TNF-α, IL-6, IL-1β, IL-18) (*P* < 0.05). (**f**)-(**i**)(**l**): ML385 treatment partially reversed the inhibitory effect of 4-OI on the mRNA and protein expression levels of NLRP3 and caspase-1 (*P* < 0.05). (**j**)-(**m**)(**l**): The expression levels of Nrf2/HO-1were inhibited by ML385 in the inhibition group (*P* < 0.05). (**n**)-(**o**): In the supernatant, the MDA level increased and the SOD activity reduced obviously in the inhibition group (*P* < 0.01). (**p**): 4-OI inhibited the ROS-positive cells during the inflammatory model induction for BMDM and ML385 partially reversed the effect. Scale bar = 50 μm, *n* = 3. ROS, reactive oxygen species. BMDM, bone marrow derived macrophage. *P < 0.05, **P < 0.01, ***P < 0.001, the model group vs. the control group. ^#^ *P *< 0.05, ^##^ *P *< 0.01, ^###^ *P *< 0.001, the model group vs. the intervention group. ns, *P* > 0.05, the model group vs. the inhibition group or the ML385 group vs. the control group

4-OI treatment significantly alleviated oxidative stress in BMDMs, as evidenced by significantly reduced MDA levels (*P* < 0.001) and ROS-positive cells, along with increased SOD activity (*P* < 0.05, Figs. 5p-q and 6p) in the intervention groups compared to the model group. The model group showed elevated MDA and SOD activity relative to the control group (*P* < 0.05), indicating an activated antioxidant response against LPS/IFN-γ-induced oxidative damage.

#### 4-OI Exerts Anti-Inflammatory and Antioxidant Effects Partially Via Nrf2/HO-1 Pathway

4-OI treatment significantly activated the Nrf2/HO-1 pathway, as demonstrated by increased protein expression of Nrf2 and HO-1 compared to the model group (*P* < 0.05, Fig. 5k, n, o). This activation was effectively blocked by the Nrf2 inhibitor ML385. In the inhibition group, the protein levels of Nrf2 and HO-1 were significantly lower than in the intervention group (*P* < 0.05), and were comparable to those in the model group (*P* > 0.05, Fig. 6k-m). A similar trend was observed at the mRNA level of HO-1 (Figs. 5m and 6j).

4-OI inhibited the M1 polarization of macrophages, and the effects were largely abolished by ML385. The protein expression of iNOS, the number of M1 macrophages, and the mRNA levels of pro-inflammatory cytokines (TNF-α, IL-6, IL-1β, IL-18) were all significantly higher in the inhibition group than in the intervention group (*P* < 0.05), and were restored to levels comparable to the model group (*P* > 0.05, Figs. 4 and 6a-e and l).

4-OI inhibited NLRP3 inflammasome activation and ML385 partially reversed the suppressive effect. The mRNA and protein expression of NLRP3 and caspase-1 were significantly increased in the inhibition group compared to the intervention group (*P* < 0.05). The expression levels in the inhibition group were comparable to those in the model group (*P* > 0.05, Fig. 6f-i, l).

4-OI inhibited oxidative stress, which was also largely reversed by ML385. The inhibition group exhibited significantly higher MDA levels and ROS-positive cells, along with lower SOD activity, compared to the intervention group (*P* < 0.01), restoring these oxidative stress parameters to levels comparable to the model group (*P* > 0.05, Fig. 6n-p).

## Discussion

The study demonstrated that 4-OI effectively mitigated acute-phase neurological injury in EAN rats. In vivo, 4-OI significantly reduced inflammatory infiltration and demyelination in the sciatic nerves, suppressed M1 macrophage polarization, inhibited pro-inflammatory cytokine release, and attenuated NLRP3 inflammasome activation and oxidative stress, primarily through the Nrf2/HO-1 signaling pathway. Consistent with these in vivo observations, in vitro experiments using BMDMs confirmed that 4-OI suppressed M1 polarization, NLRP3 inflammasome activity, inflammatory mediator production, while enhancing antioxidant responses through Nrf2/HO-1 activation. These effects were partially reversed by the Nrf2 inhibitor ML385, collectively indicating that 4-OI confers neuroprotection through dual modulation of inflammatory and oxidative stress pathways.

The pivotal role of macrophages in immune-mediated demyelination is well-established in GBS and its animal models. Autopsy studies of AIDP, a major GBS subtype, consistently show inflammatory infiltrates comprising of T cells and macrophages, which colocalize with sites of segmental demyelination and complement deposition on Schwann cells [17–19]. Macrophages contribute to peripheral nerve injury through multiple mechanisms, including T cell activation, release of reactive oxygen species (ROS) and matrix metalloproteinase-9, and direct myelin sheath invasion [18]. The predominance of M1-polarized macrophages during the acute phase of EAN further underscores their critical involvement in disease pathogenesis.

A central mediator of macrophage-driven inflammation is the NLRP3 inflammasome, a multiprotein complex assembled by pyrin–pyrin domain interactions between NLRP3 and the adaptor ASC, which recruits and activates caspase-1 [20–22]. Activated caspase-1 cleaves pro-IL-1β and pro-IL-18 into their mature forms, amplifying local inflammatory response. Beyond cytokine maturation, NLRP3 inflammasome activation promotes metabolic reprogramming and upregulation of surface molecules in macrophages, reinforcing their M1-polarized state and pro-inflammatory capacity [23–25].

Oxidative stress represents another critical pathogenic mechanism in GBS, directly contributing to demyelination and disruption of the blood-nerve barrier. Lipid peroxidation, driven by oxygen free radicals, damages membrane integrity and generates pro-inflammatory lipid mediators [26, 27].Clinical evidence supports the involvement of oxidative stress, as GBS patients exhibit systemic oxidative imbalance accompanied by impaired antioxidant defenses [28–31]. The interplay between oxidative injury and immune activation is underscored by observations that ROS not only facilitates myelin phagocytosis by macrophages but is also required for NLRP3 inflammasome activation [32]. Genetic studies further substantiate this connection: a CYBA genotype associated with reduced ROS production correlates with improved clinical outcomes in GBS patients [33]. Additionally, oxidative stress is implicated in GBS cases triggered by viral infections such as Zika virus, which perturbs redox balance to promote viral replication and nerve injury [34–39] Given its central role in GBS pathogenesis, oxidative stress represents a promising therapeutic target.

Despite the promising findings, this study has several limitations. (1) The use of a single animal model may not fully recapitulate the heterogeneous clinical spectrum of human GBS. (2) Although we established that 4-OI modulates macrophage function, its precise molecular targets within these cells remain incompletely characterized. (3) While our research primarily focused on innate immunity, the potential effects of 4-OI on adaptive immune responses, warrants future investigation. Based on these limitations, we propose the following future directions: (1) validating the therapeutic efficacy of 4-OI in complementary GBS models; (2) applying proteomic strategies to systematically identify direct cellular targets of 4-OI, along with transcriptional profiling of a broader set of Nrf2-regulated genes (e.g., SLC7A11, Sestrin2) to more comprehensively define the antioxidant response; (3) exploring the immunomodulatory impact of 4-OI on macrophage–lymphocyte interactions; and (4) performing comprehensive off-target profiling and long-term toxicological studies in relevant preclinical models to support clinical translation of 4-OI. Addressing these questions will be essential for advancing 4-OI toward clinical application in GBS.

## Conclusion

4-OI ameliorated acute-phase EAN by suppressing M1 macrophage polarization, inhibiting NLRP3 inflammasome activation, and attenuating oxidative stress, partially through the Nrf2/HO-1 signaling pathway. Mechanistically, 4-OI may suppress ROS generation via Nrf2/HO-1 activation, thereby curbing NLRP3 inflammasome activity and subsequent M1 macrophage polarization. These findings highlight the potential of 4-OI as a therapeutic candidate for GBS, though further studies are needed to clarify its precise molecular targets, efficacy in complementary models, and immunomodulatory effects beyond macrophages.

## Supplementary Information

Below is the link to the electronic supplementary material.

Supplementary Material 1 (DOCX 32.2 MB)

## Data Availability

No datasets were generated or analysed during the current study.
