# Supplementary material for: The Neuroprotective Effect of 4-Octyl Itaconate on Acute Period of Experimental Autoimmune Neuritis
Source: Inflammation. 2025 Dec 17;49(1):5. doi: 10.1007/s10753-025-02370-w (PMC12711925; doi:10.1007/s10753-025-02370-w)

Raw images from western blots

1. Proteins in Sciatic nerves
2. NLRP3 CON+NS/EAN+NS/EAN+4-OI/CON+NS/EAN+NS/EAN+4-OI

(a)
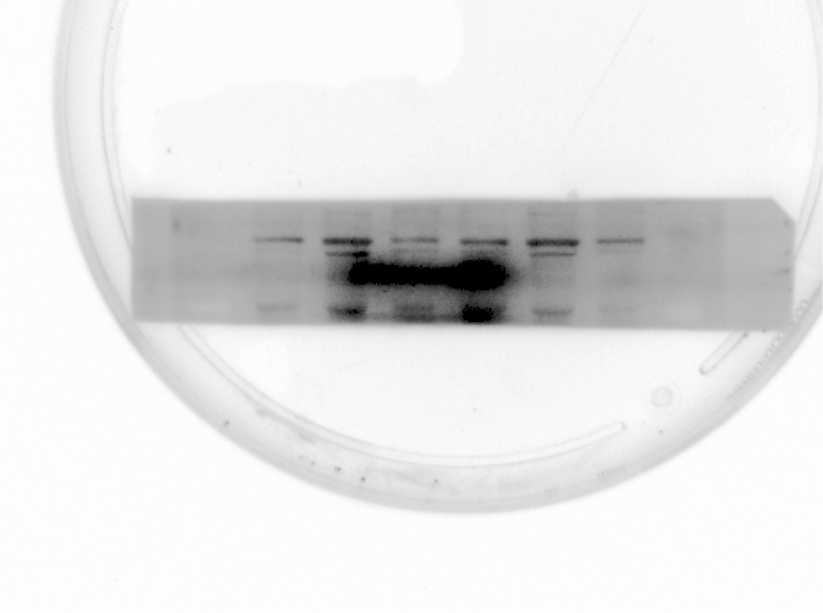
(b)
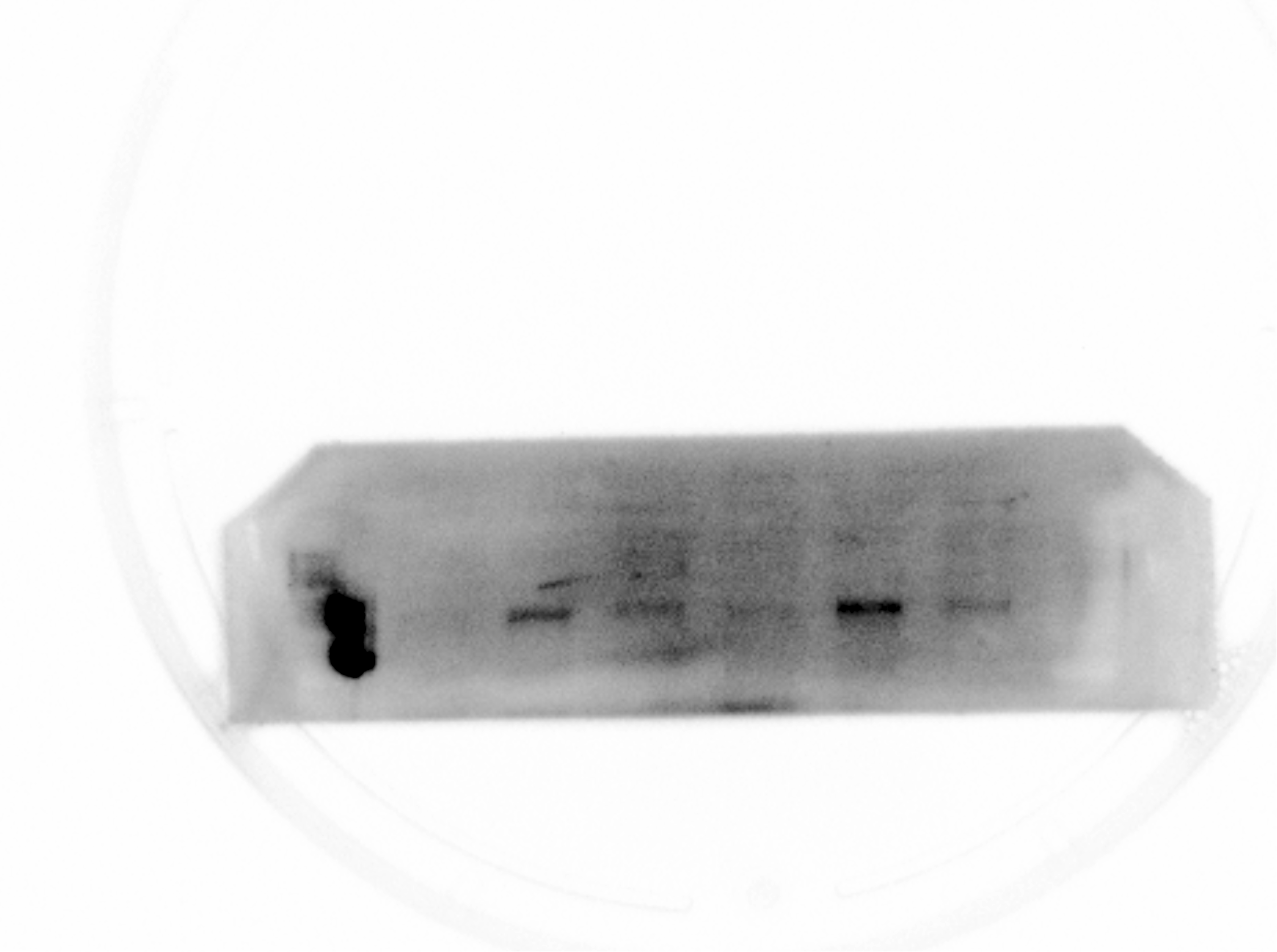


(1) Caspase-1 CON+NS/EAN+NS/EAN+4-OI/CON+NS/EAN+NS/EAN+4-OI

(a)
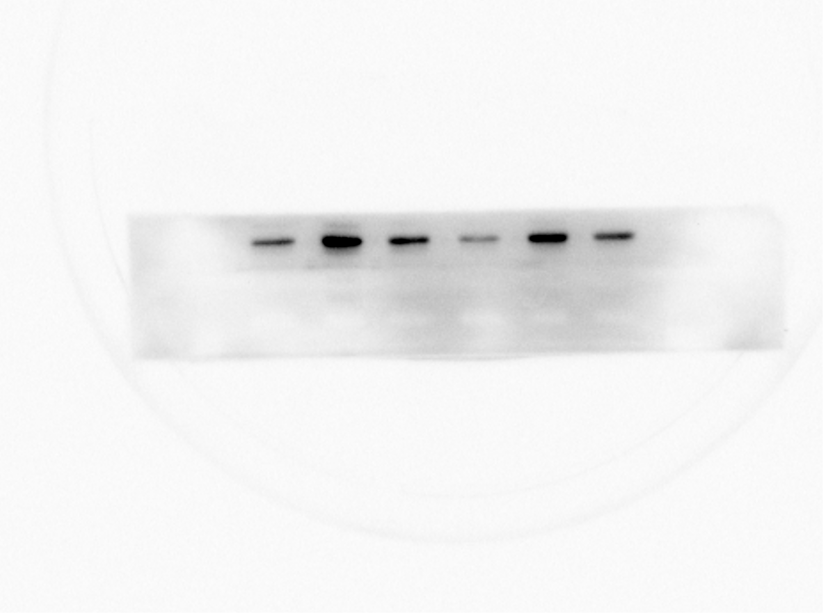
(b)
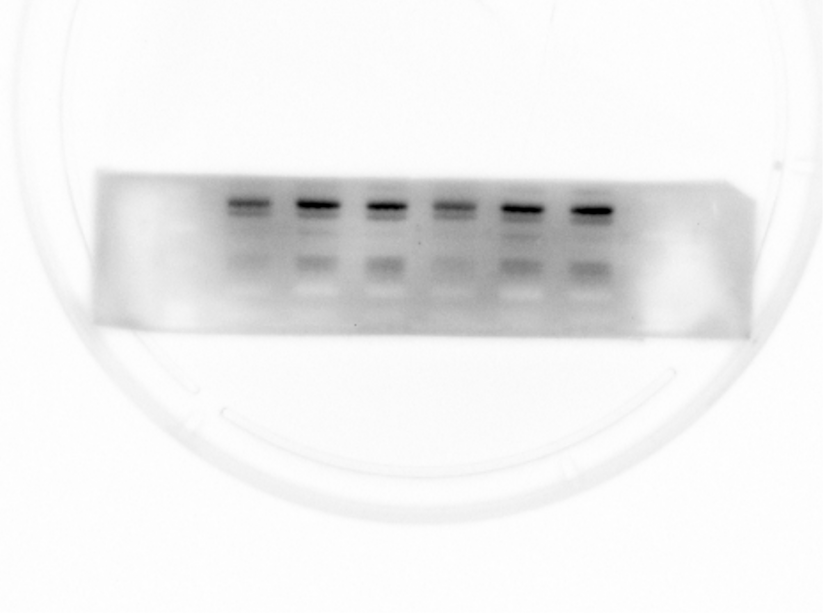


(1) β-actin CON+NS/EAN+NS/EAN+4-OI/CON+NS/EAN+NS/EAN+4-OI

(a)
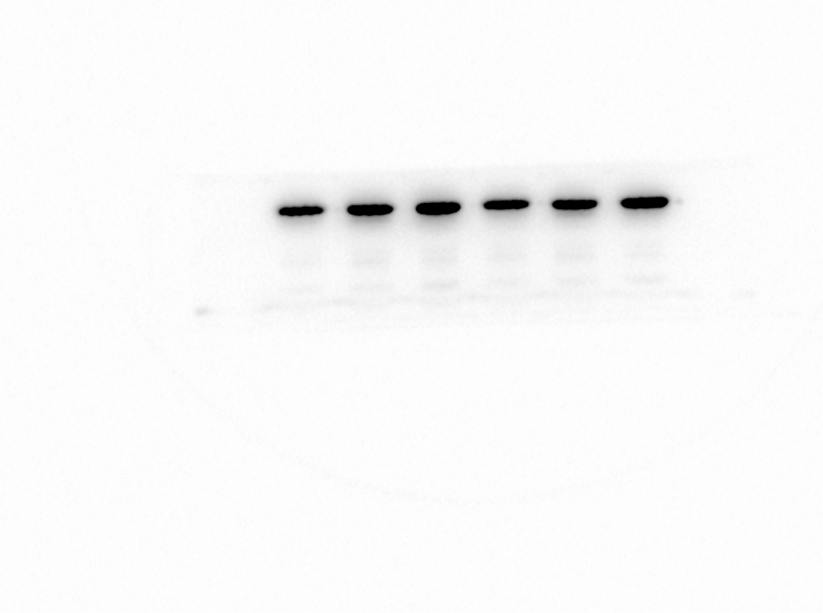
(b)
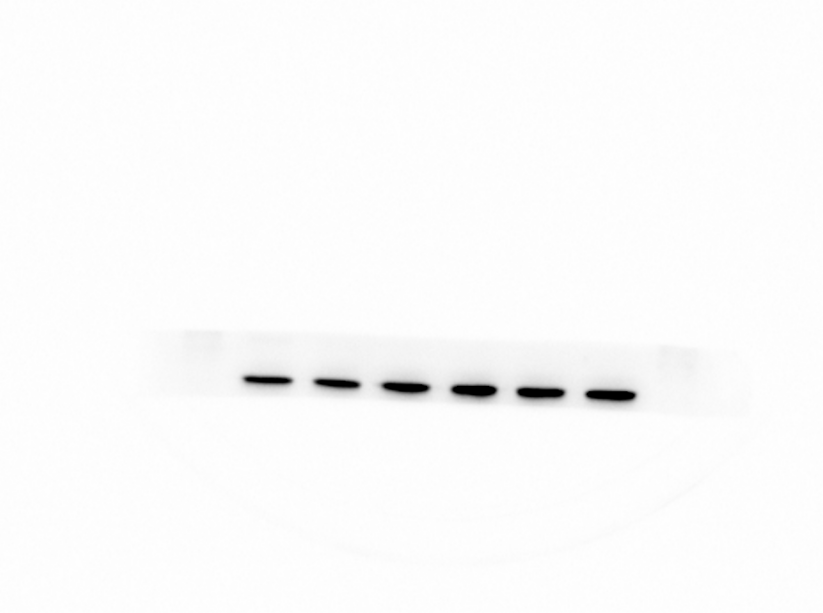


(2) ASC CON+NS/EAN+NS/EAN+4-OI CON+NS/EAN+NS/EAN+4-OI

(a)
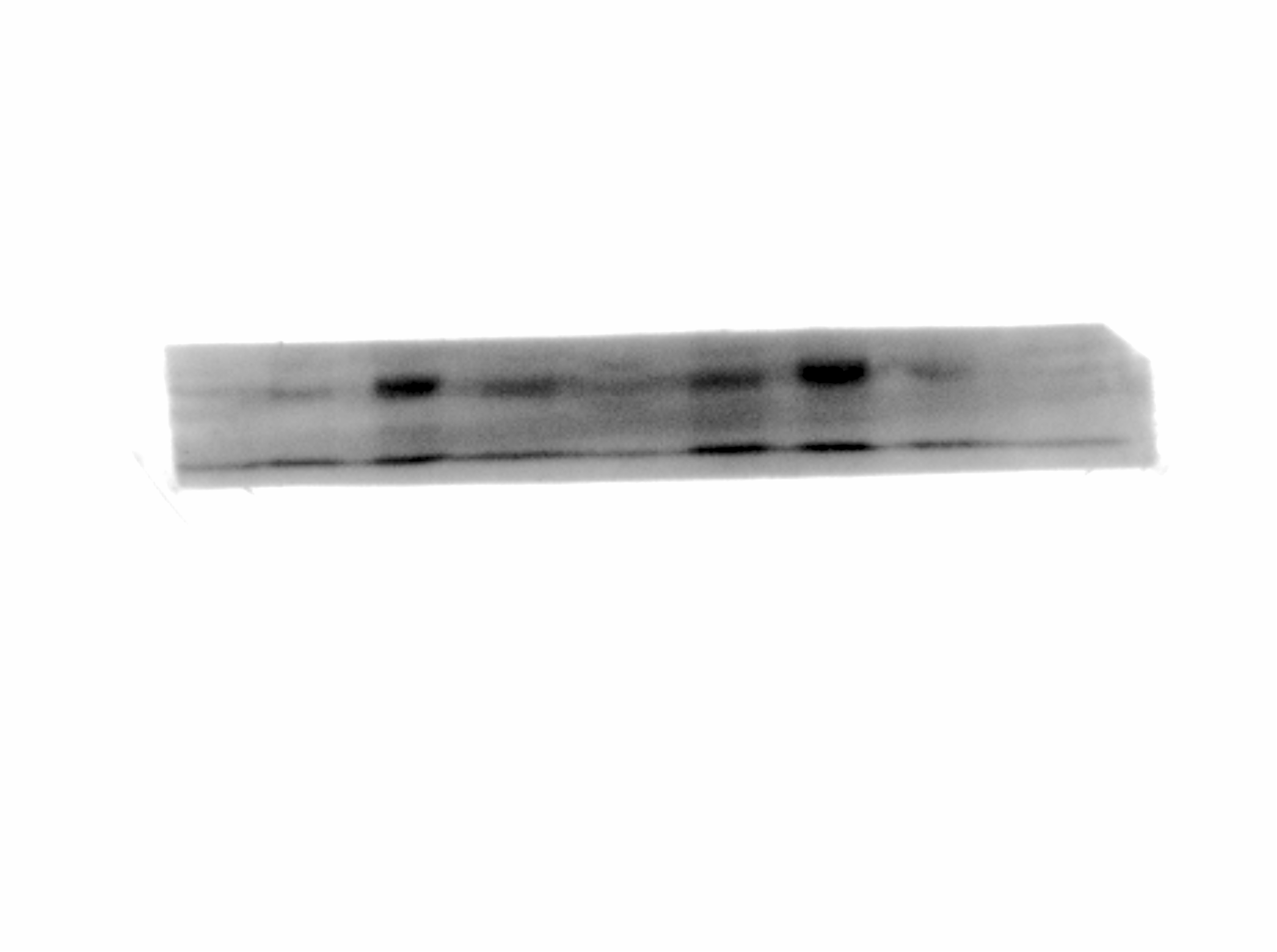
(b)
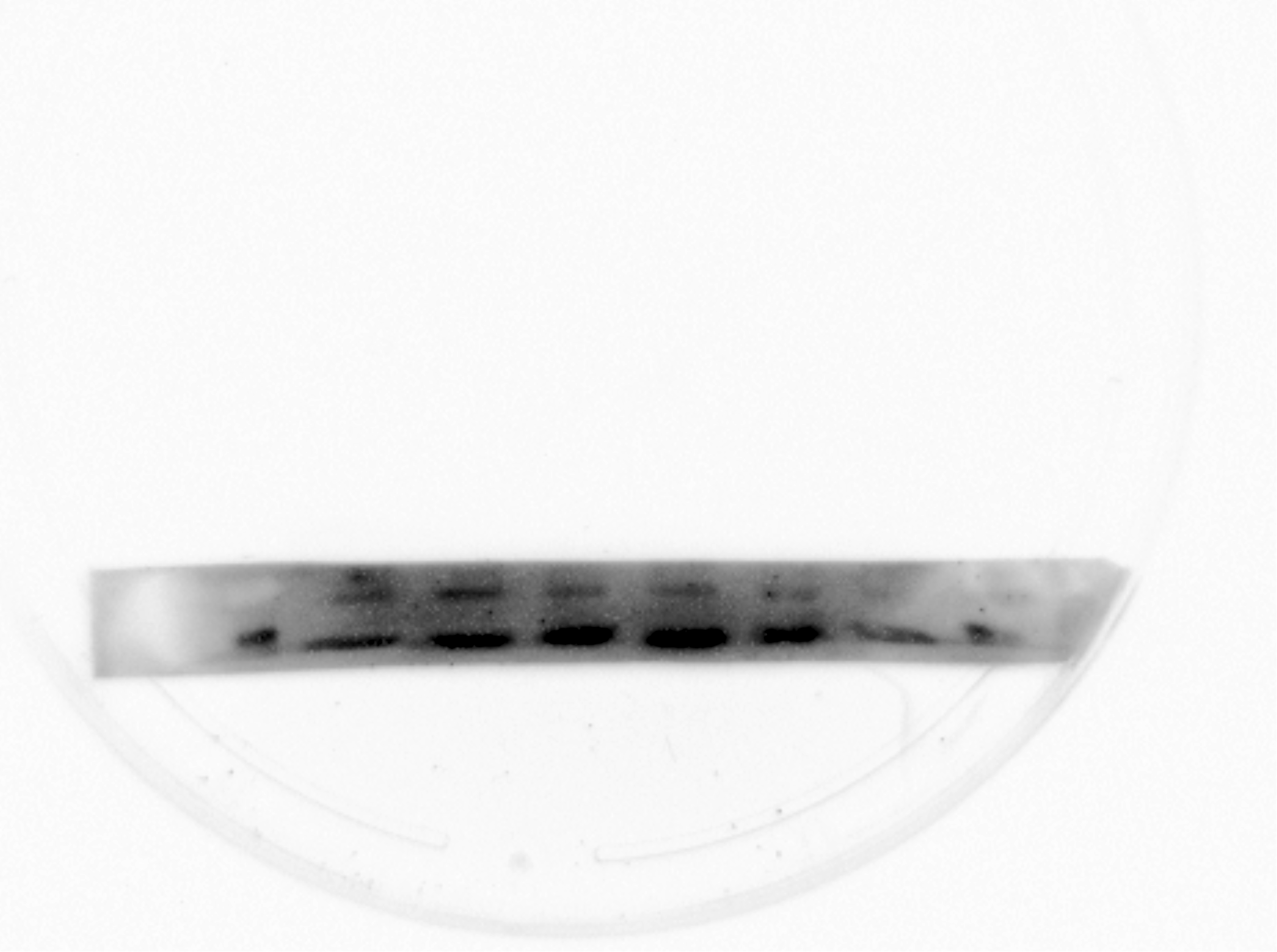


(2) β-actin CON+NS/EAN+NS/EAN+4-OI CON+NS/EAN+NS/EAN+4-OI

(a)
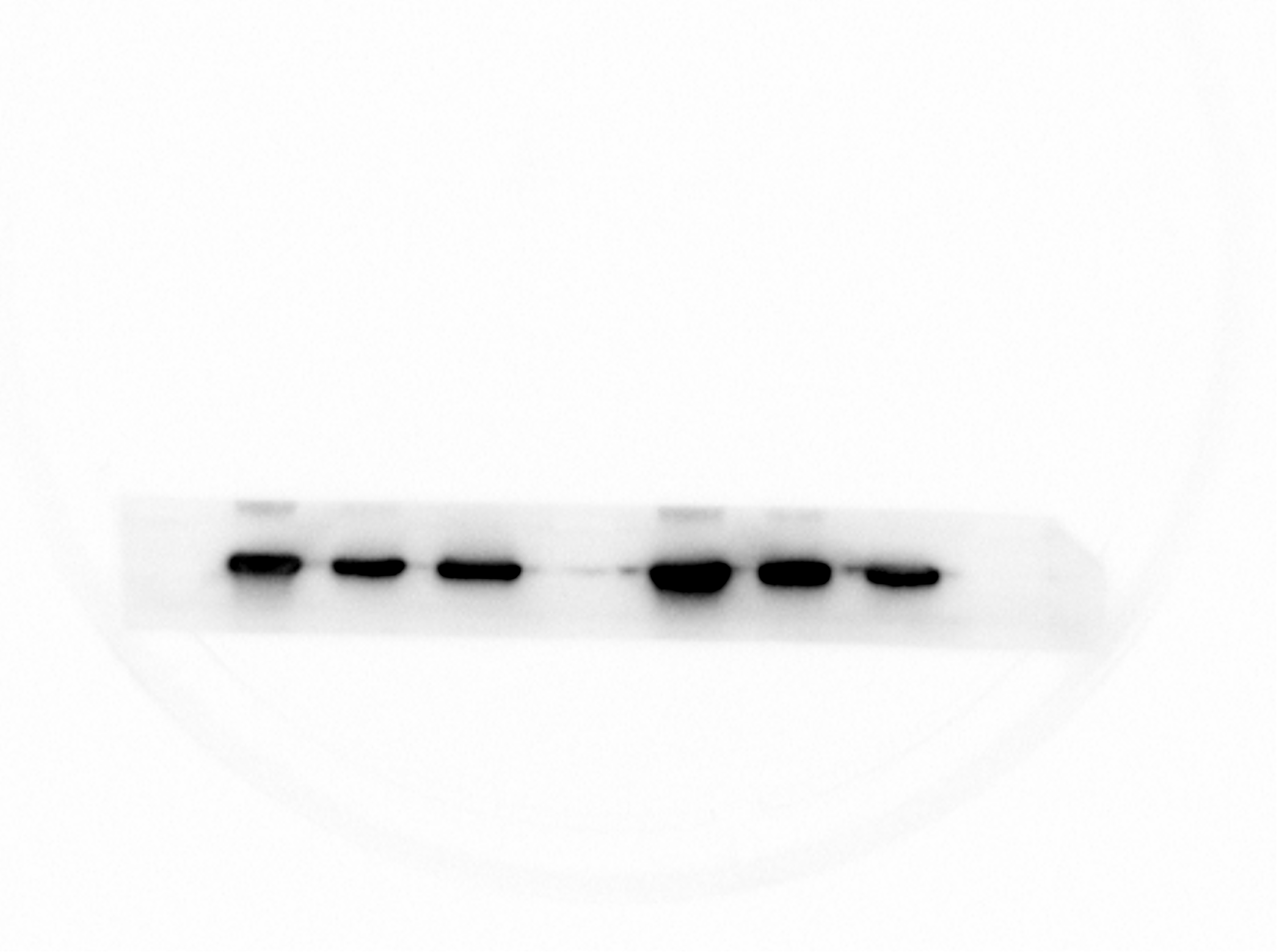
(b)
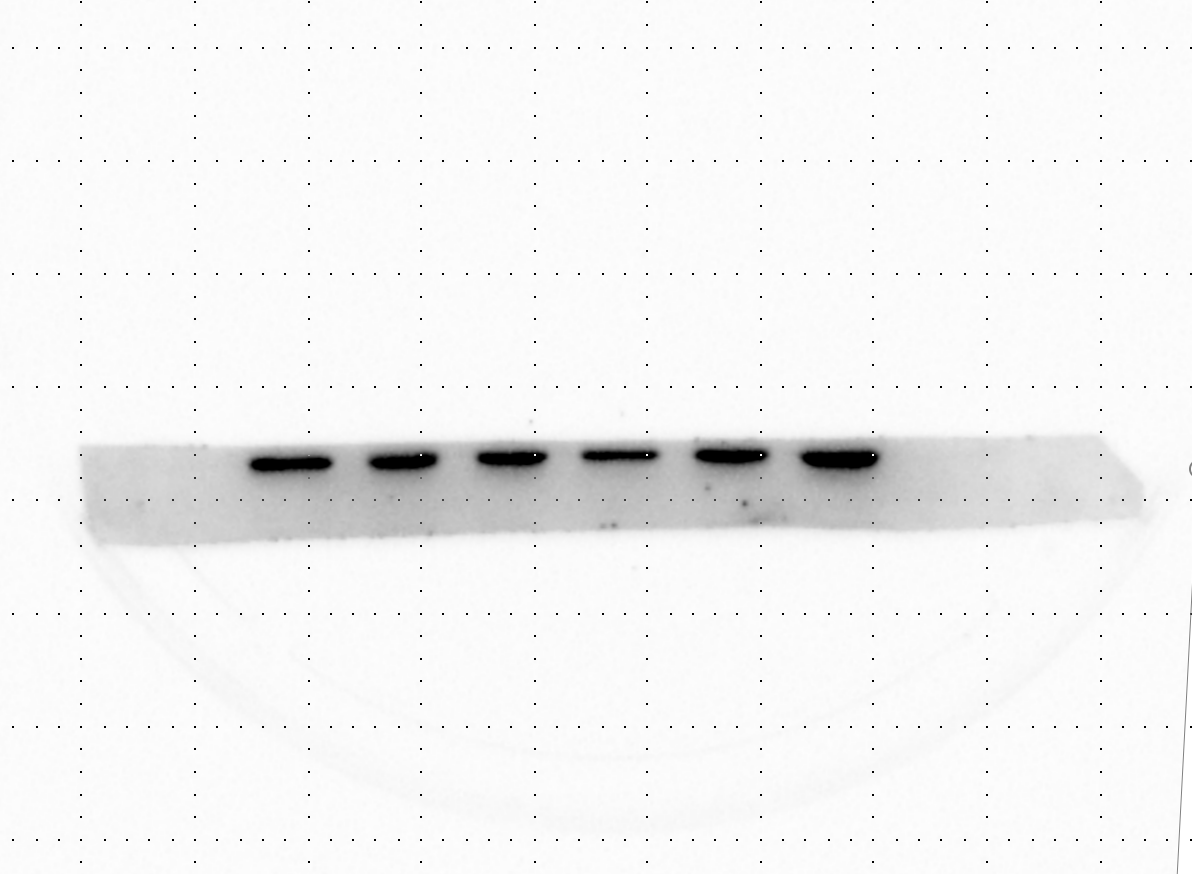


(3) Nrf2 CON+NS/EAN+NS/EAN+4-OI/CON+NS/EAN+NS/EAN+4-OI

(a)
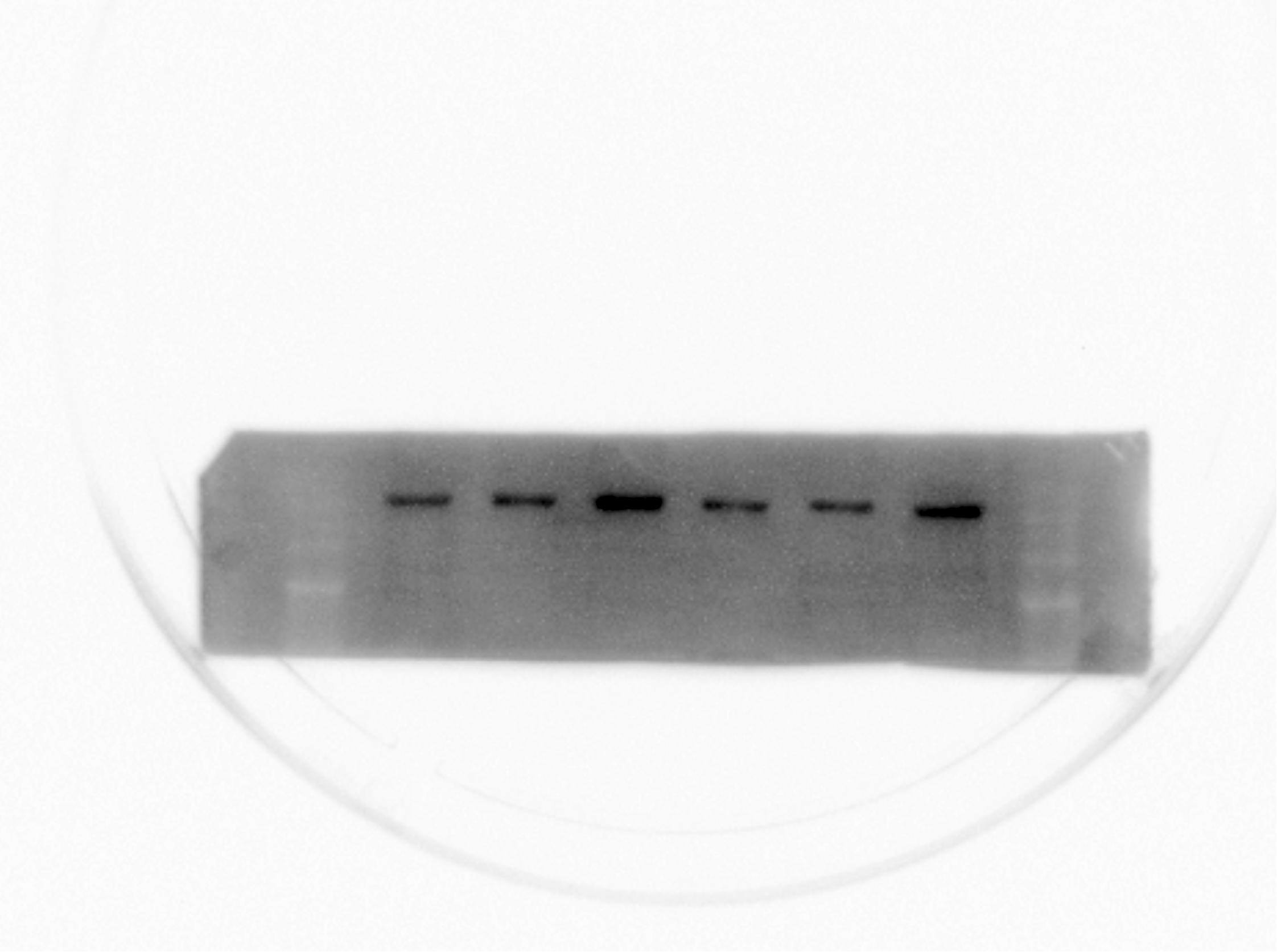
(b)
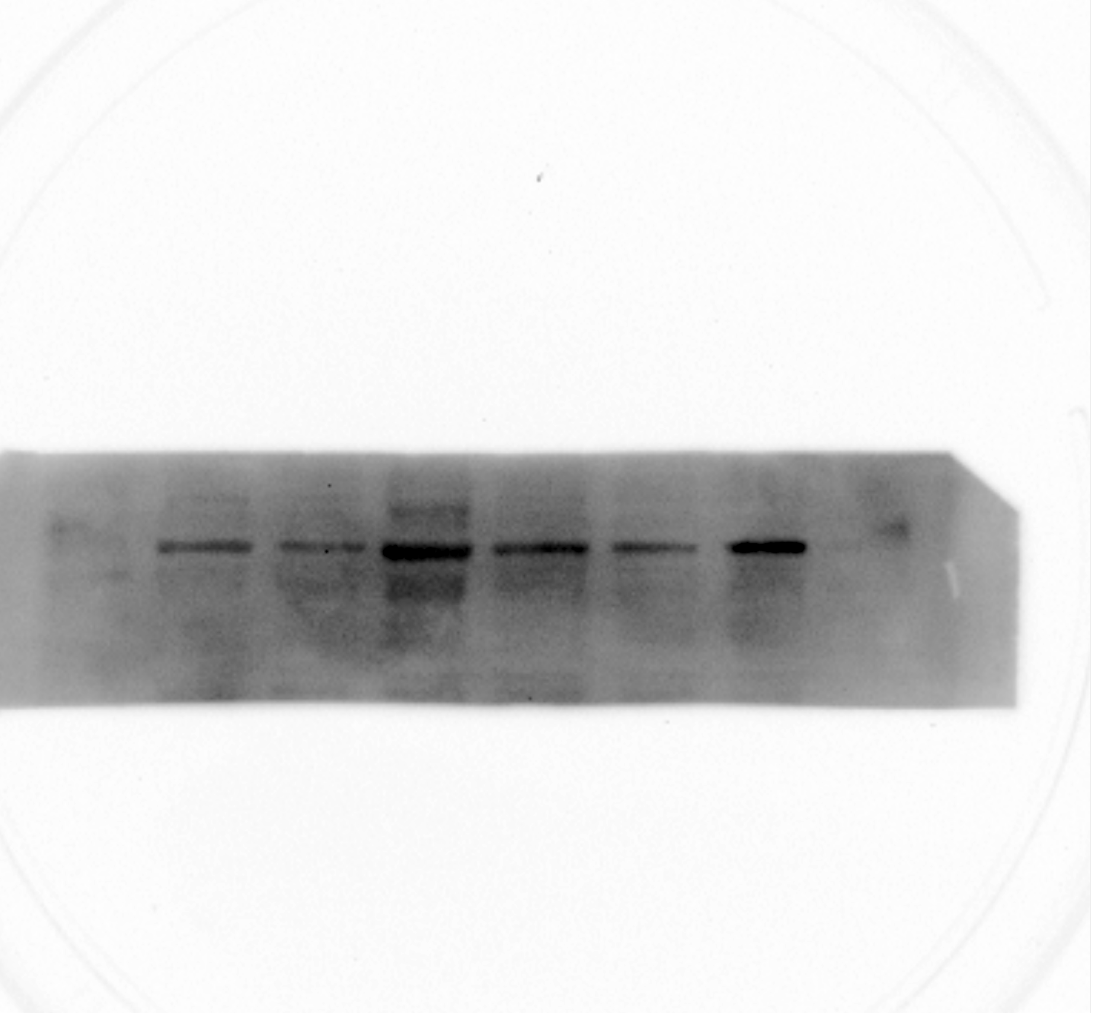


(3) β-actin CON+NS/EAN+NS/EAN+4-OI/CON+NS/EAN+NS/EAN+4-OI

(a)
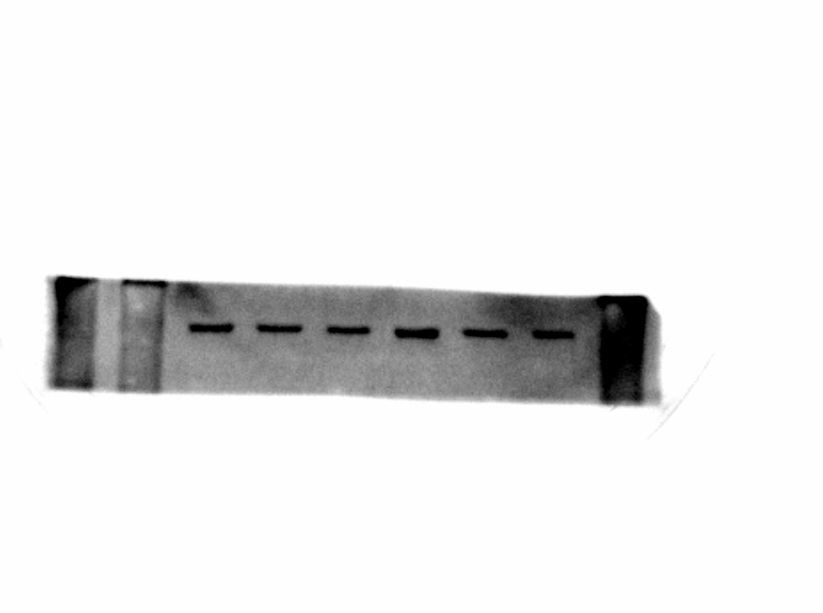
 (b)
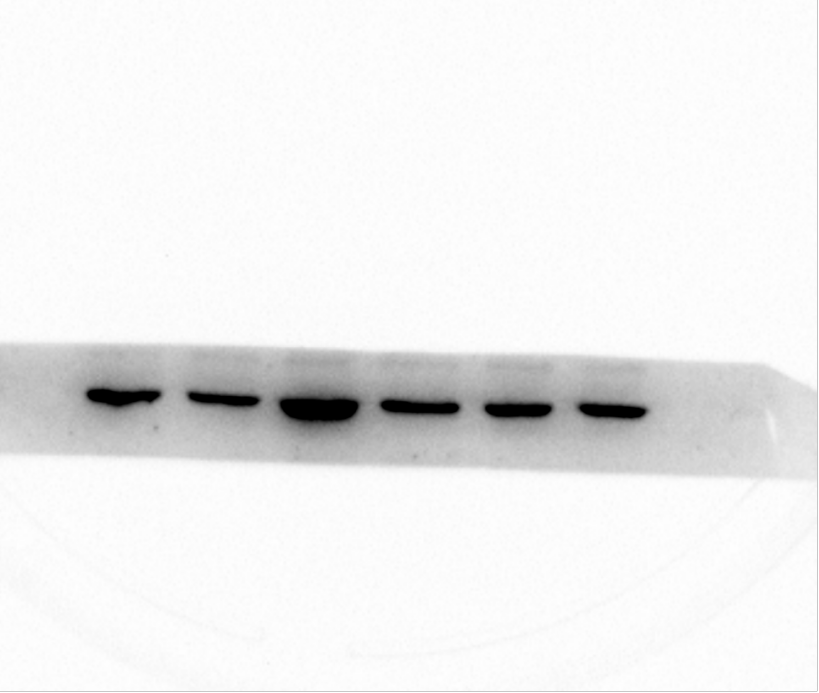


(4) HO-1 CON+NS/EAN+NS/EAN+4-OI CON+NS/EAN+NS/EAN+4-OI


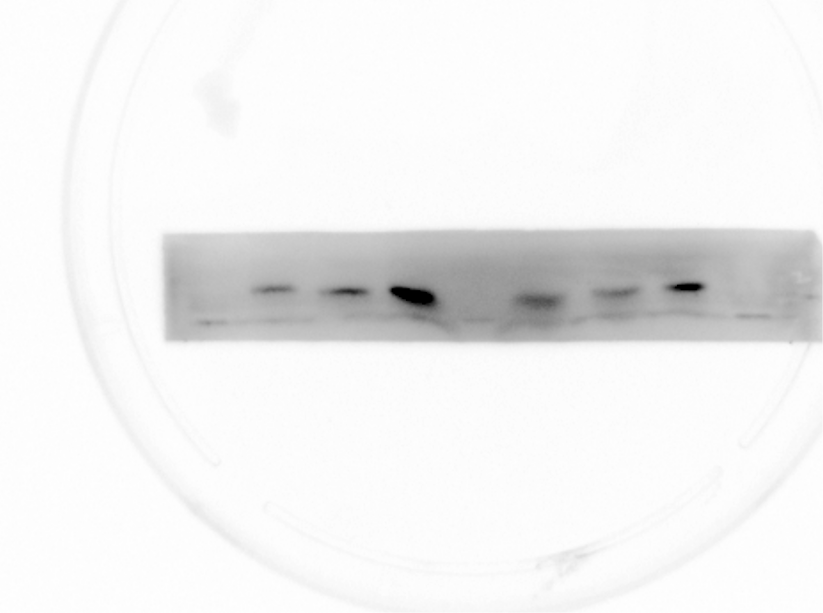


(4) β-actin CON+NS/EAN+NS/EAN+4-OI CON+NS/EAN+NS/EAN+4-OI


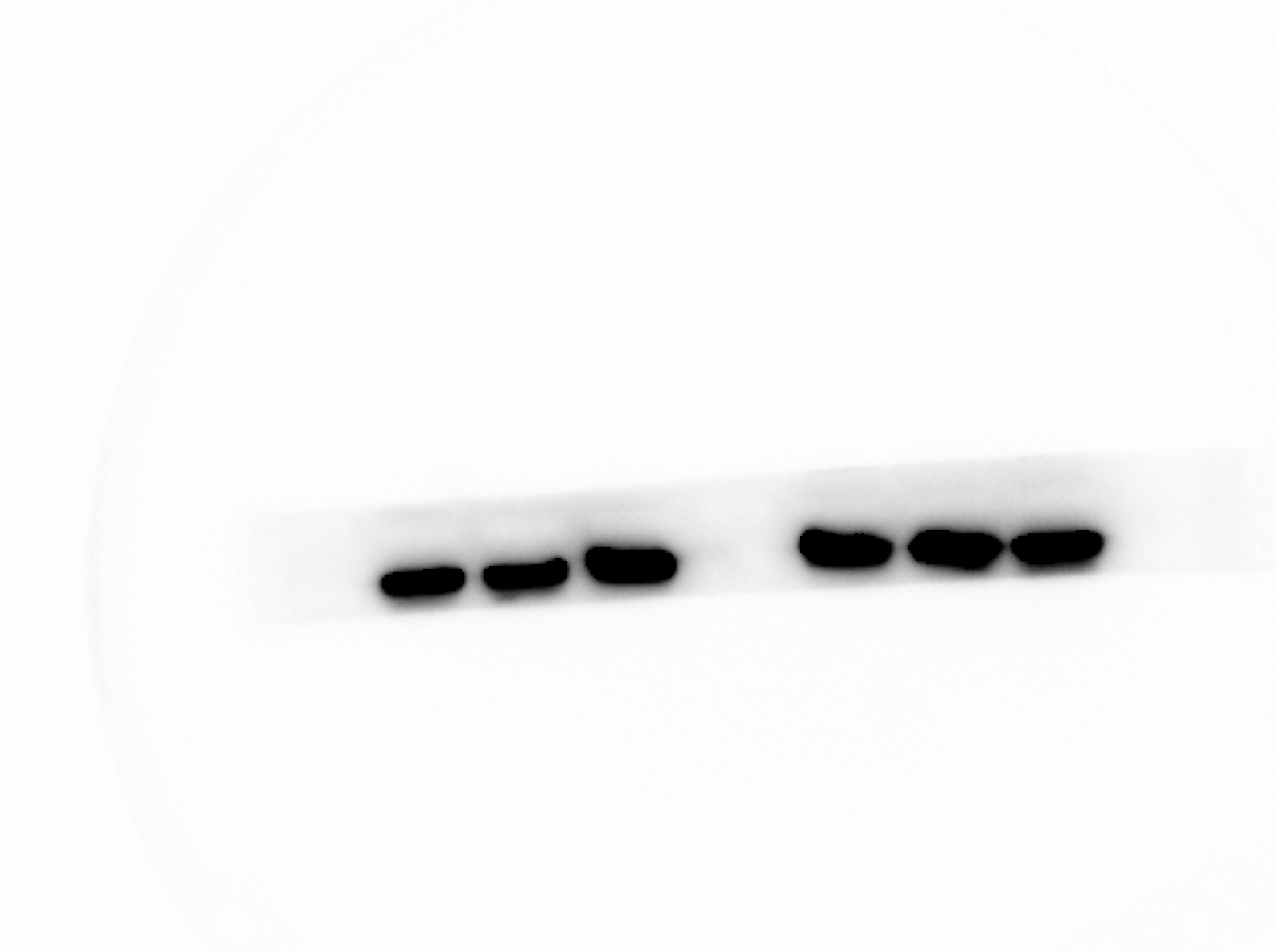


(5) HO-1 CON+NS/EAN+NS/EAN+4-OI/CON+NS/EAN+NS/EAN+4-OI


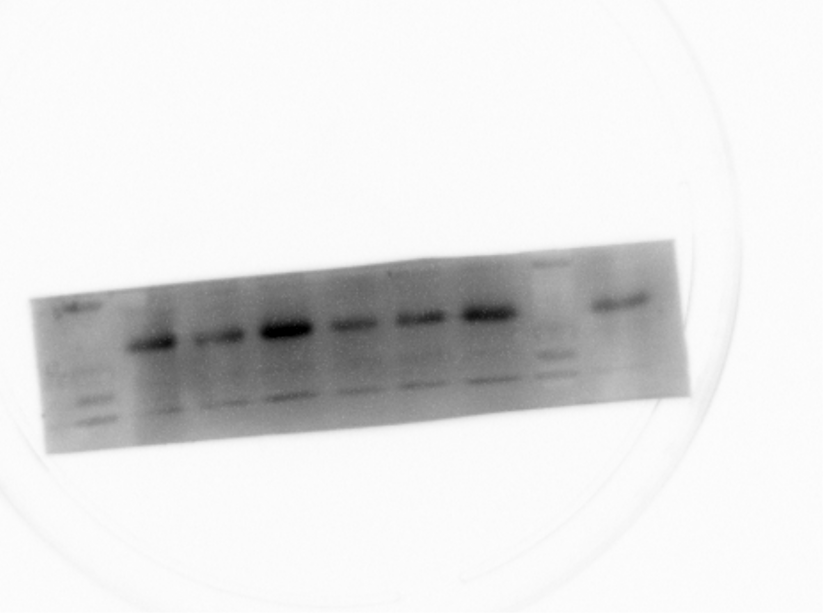


(5) β-actin CON+NS/EAN+NS/EAN+4-OI/CON+NS/EAN+NS/EAN+4-OI


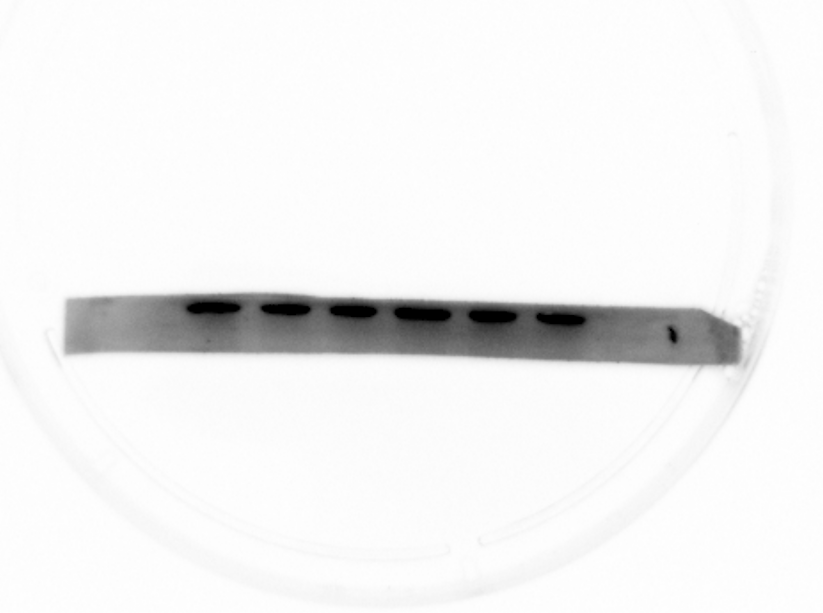


1. Proteins in BMDM (con/con+4-OI/LPS+IFN-γ/125μM4-OI+LPS+IFN-γ/250μM4-OI+LPS+IFN-γ;

con/con+mL385/LPS+IFN-γ/250 μM 4-OI+LPS+IFN-γ/250 μM 4-OI+ML385+LPS+IFN-γ)

1. iNOS

（a）
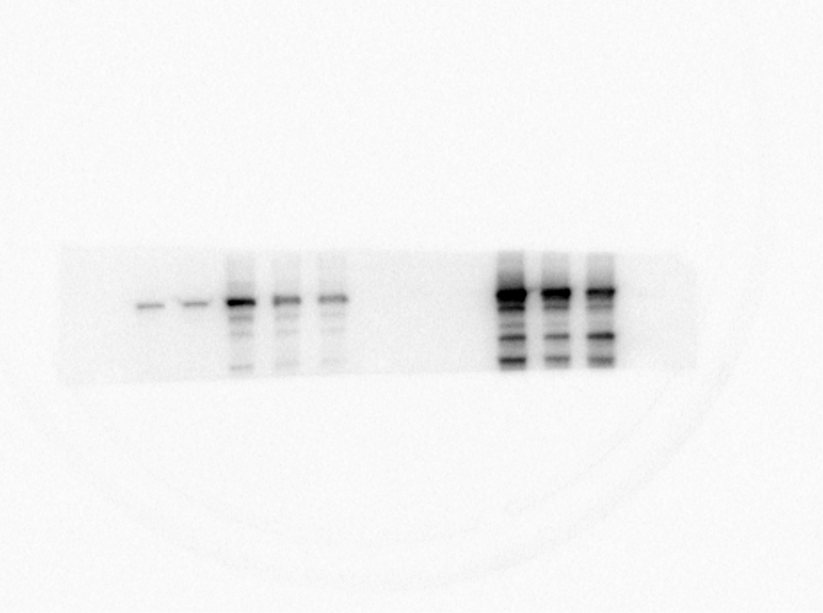
(b)
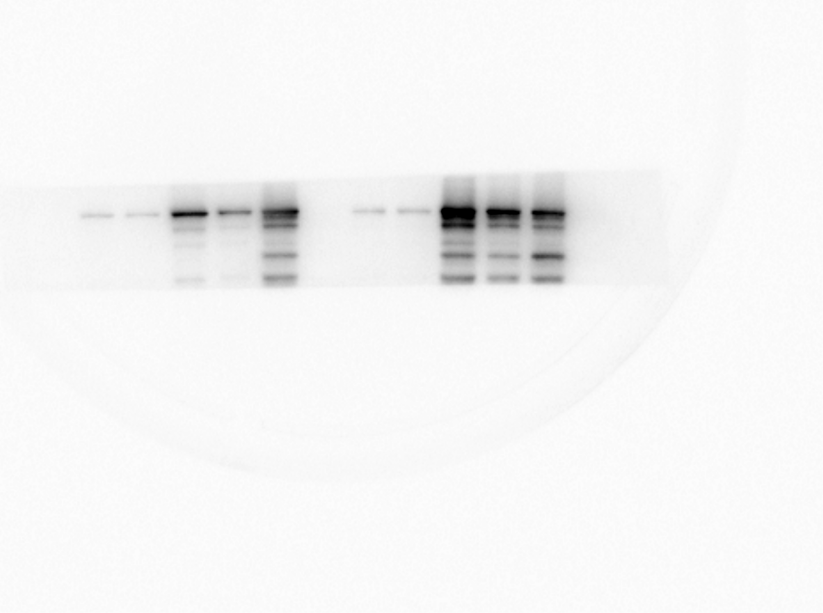


(c)
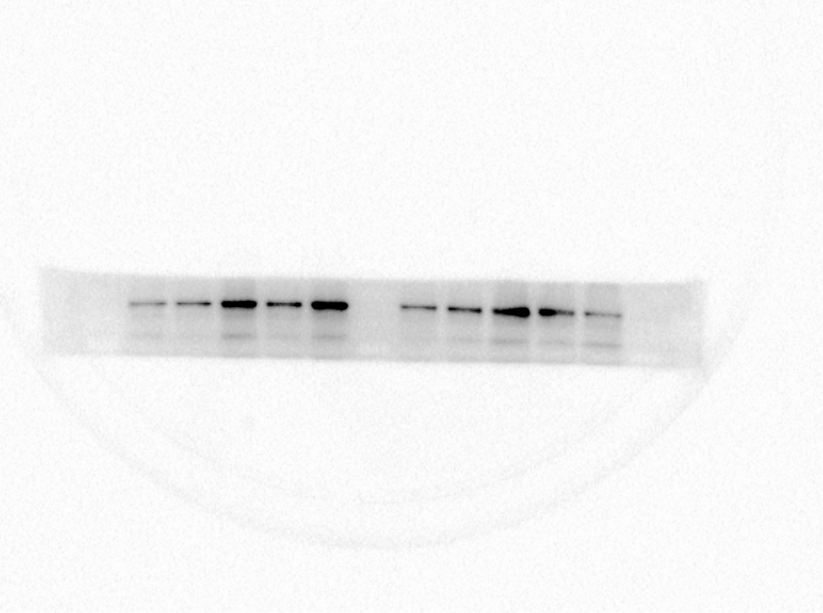
(d)
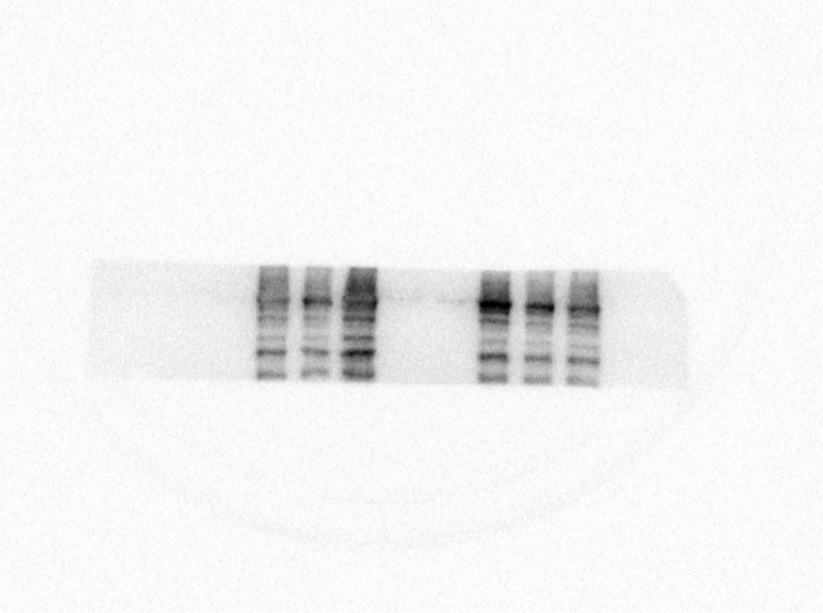


(1) β-actin

(a)
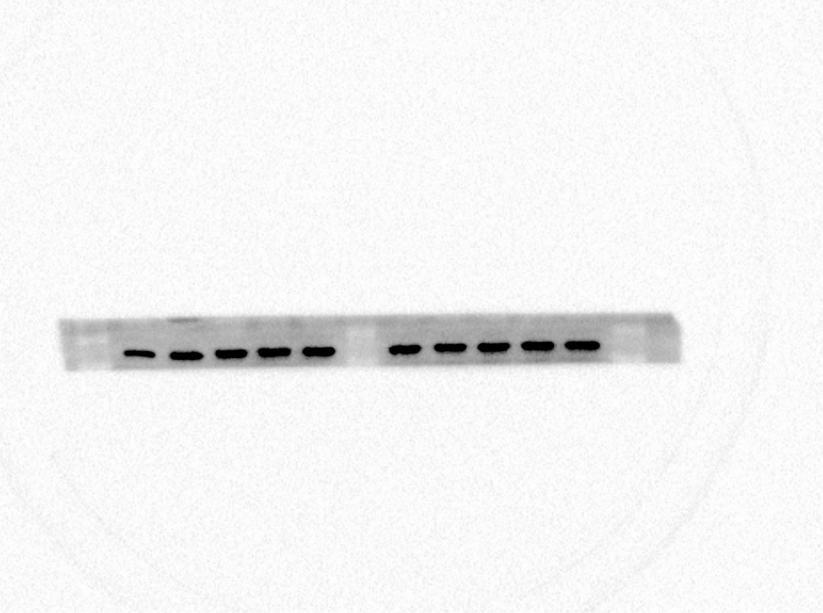
(b)
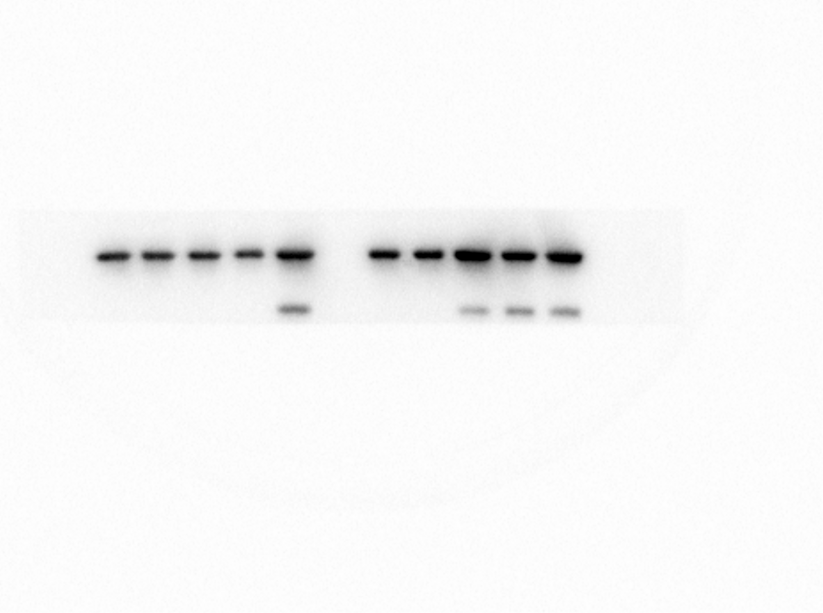


(c)
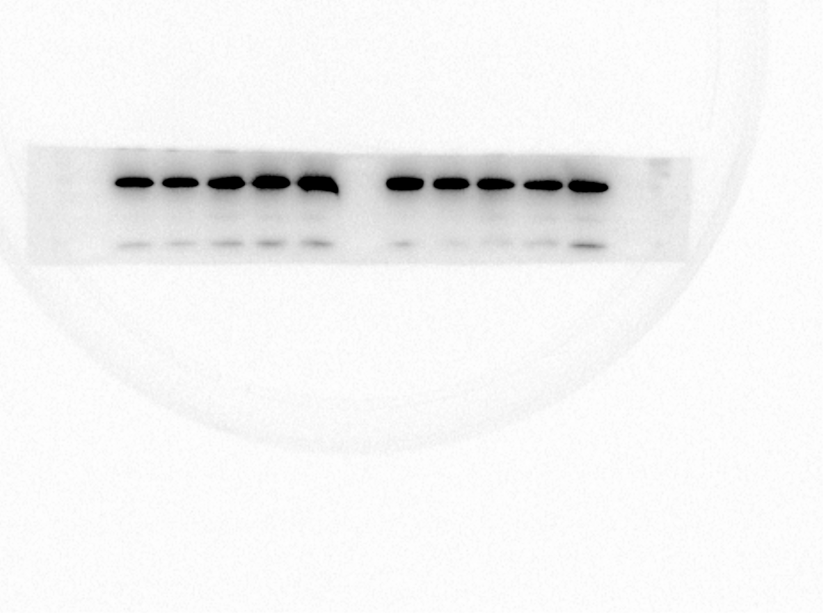
(d)
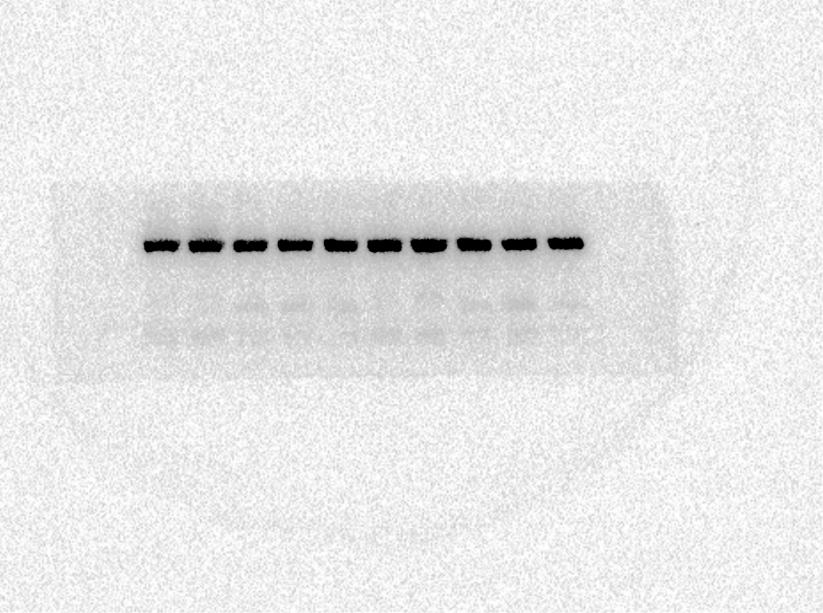


1. NLRP3
2.
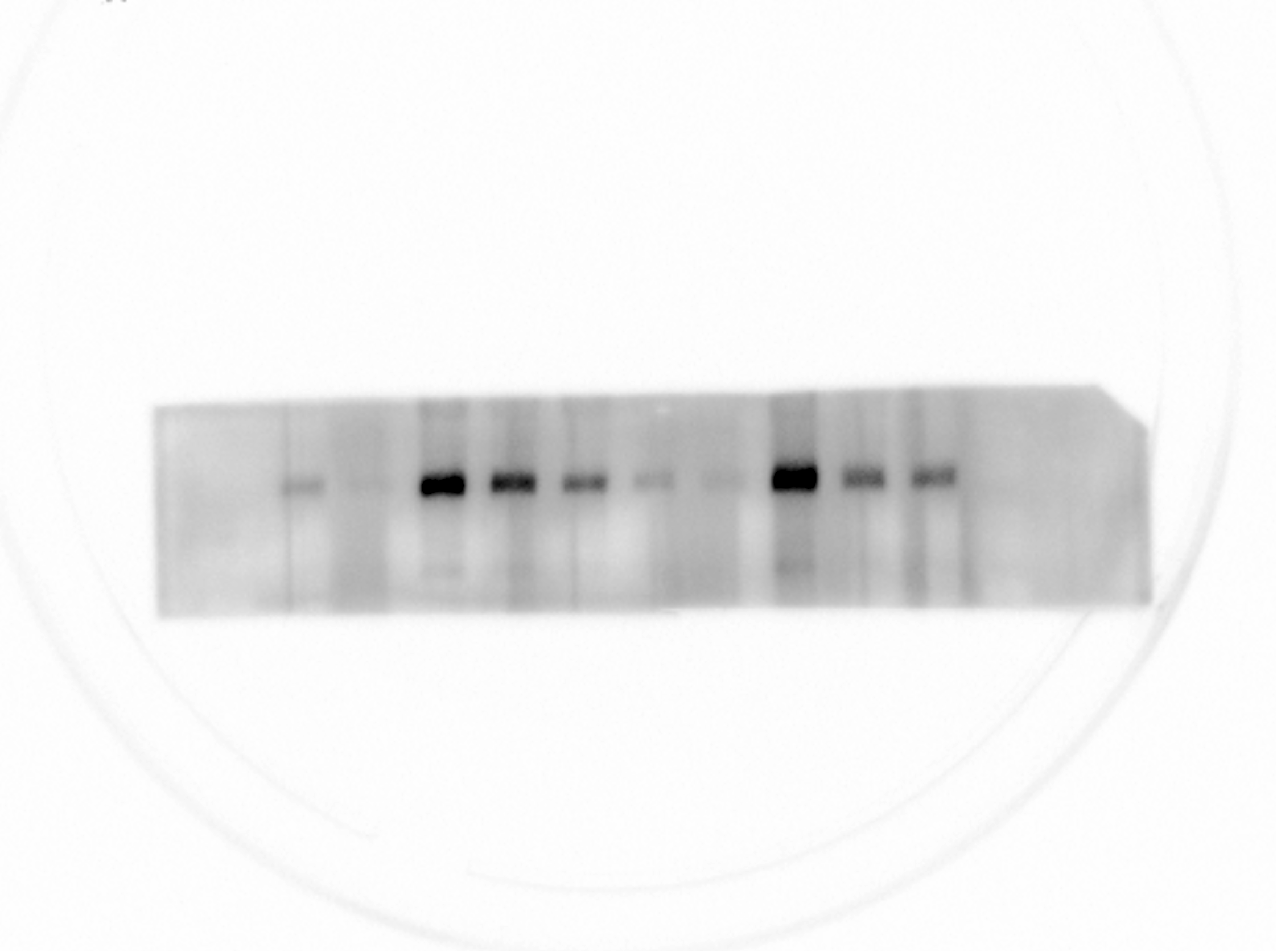
 (b)
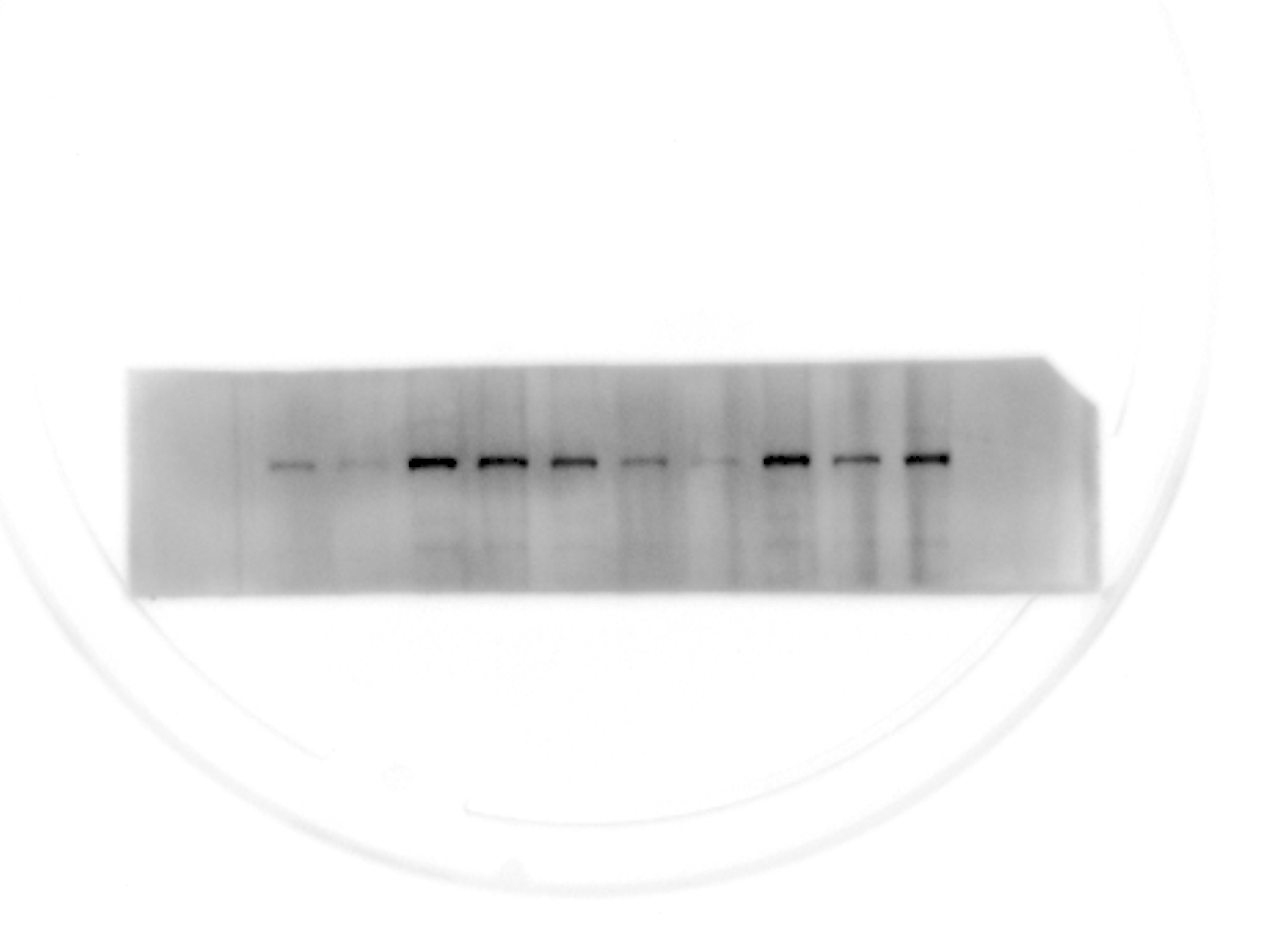

3.
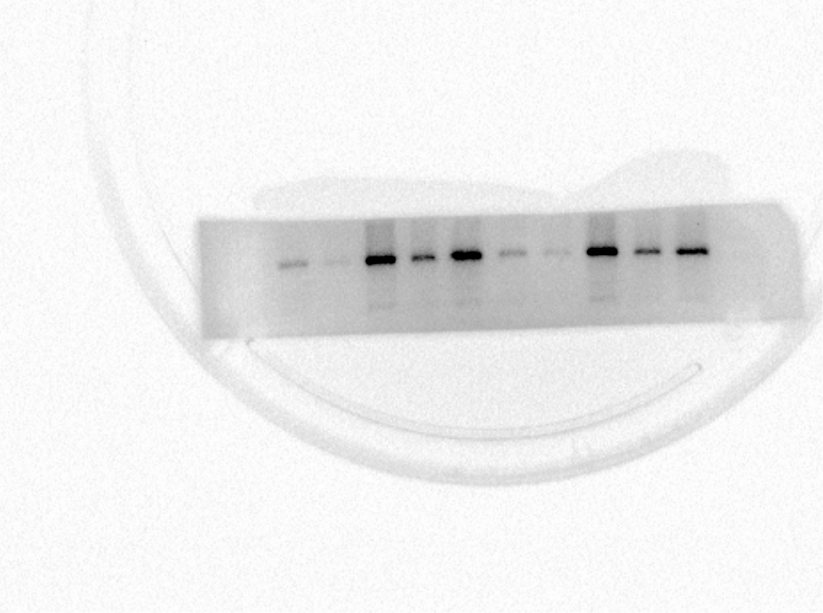


(2) β-actin

(a)
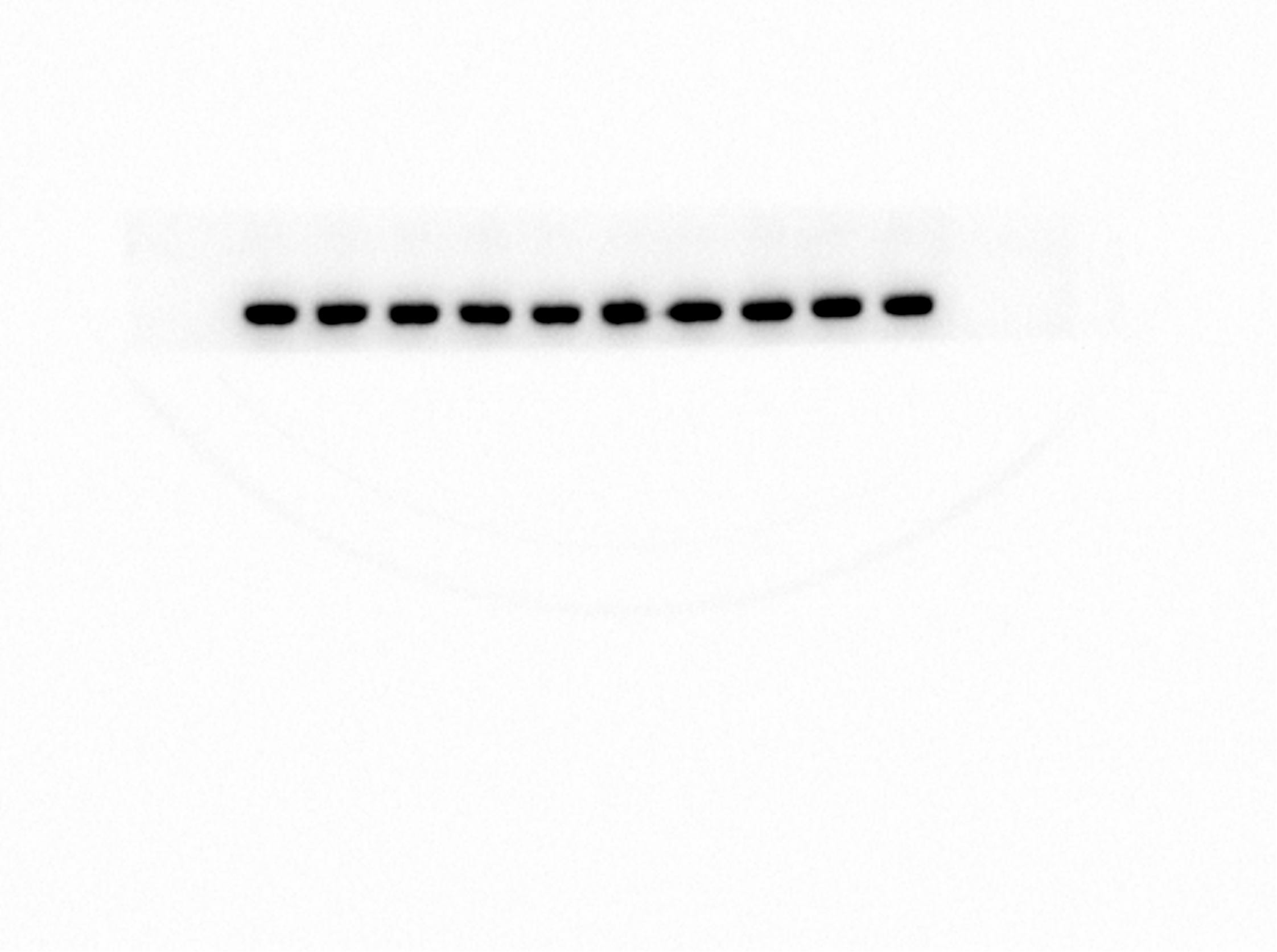
(b)
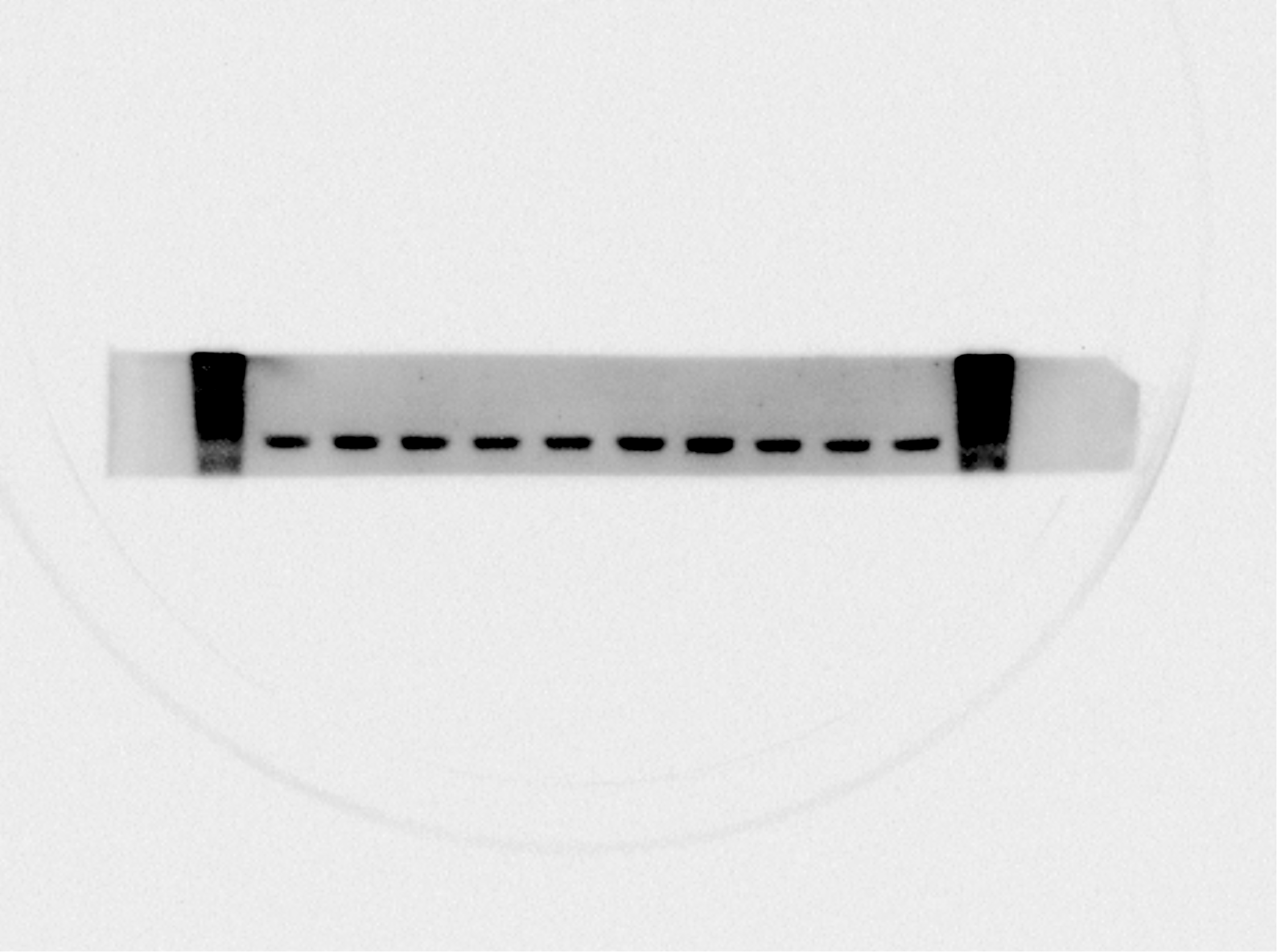


(c)
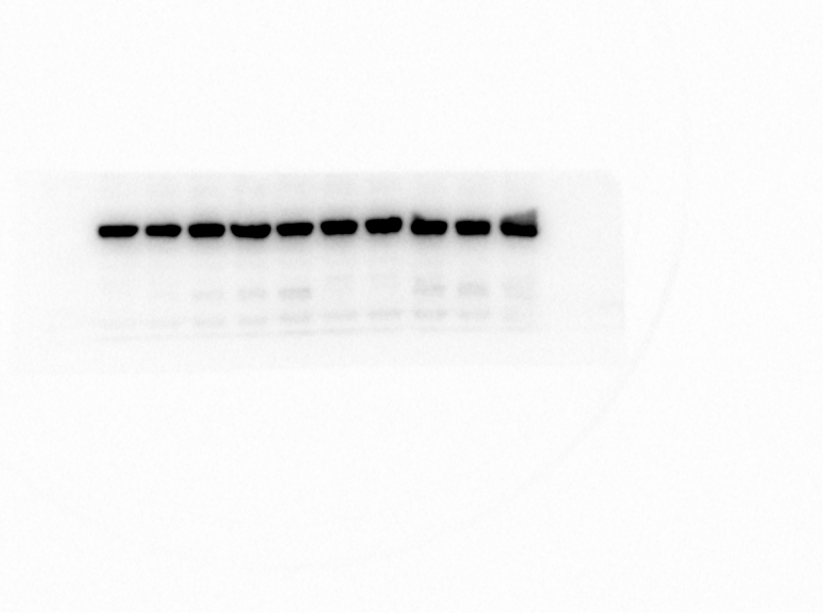


(3) Caspase-1

(a)
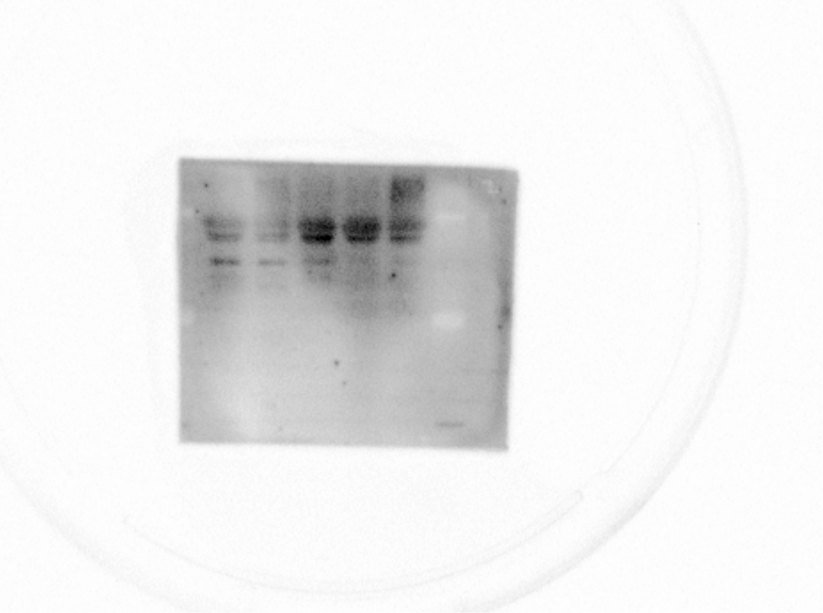
(b)
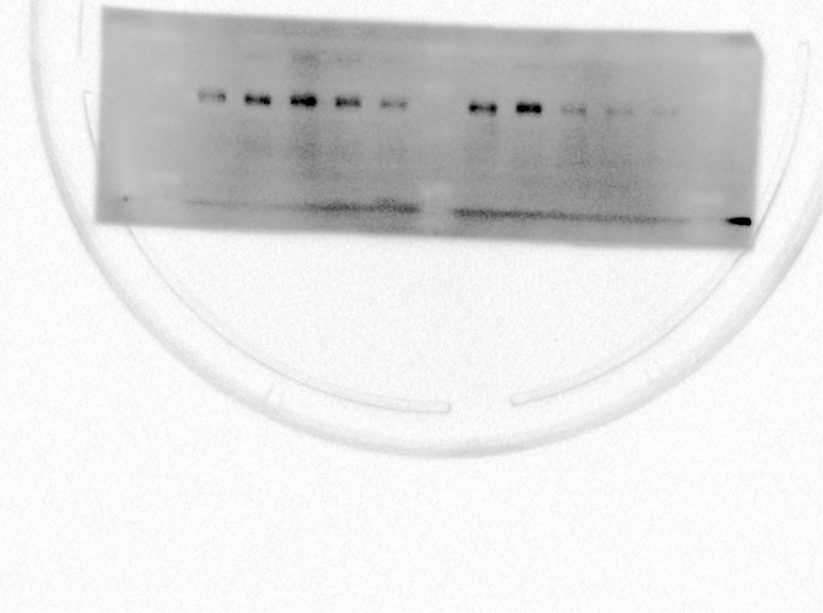
(c)
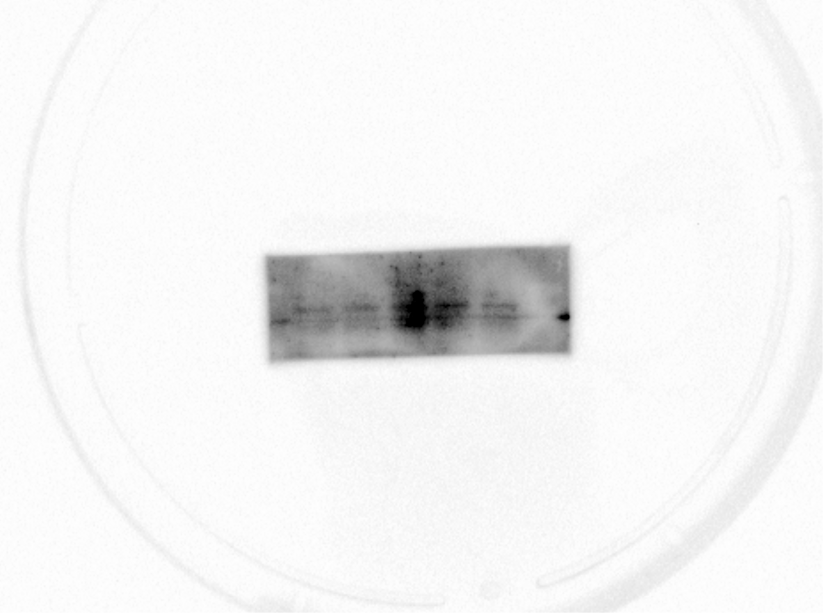


(d)
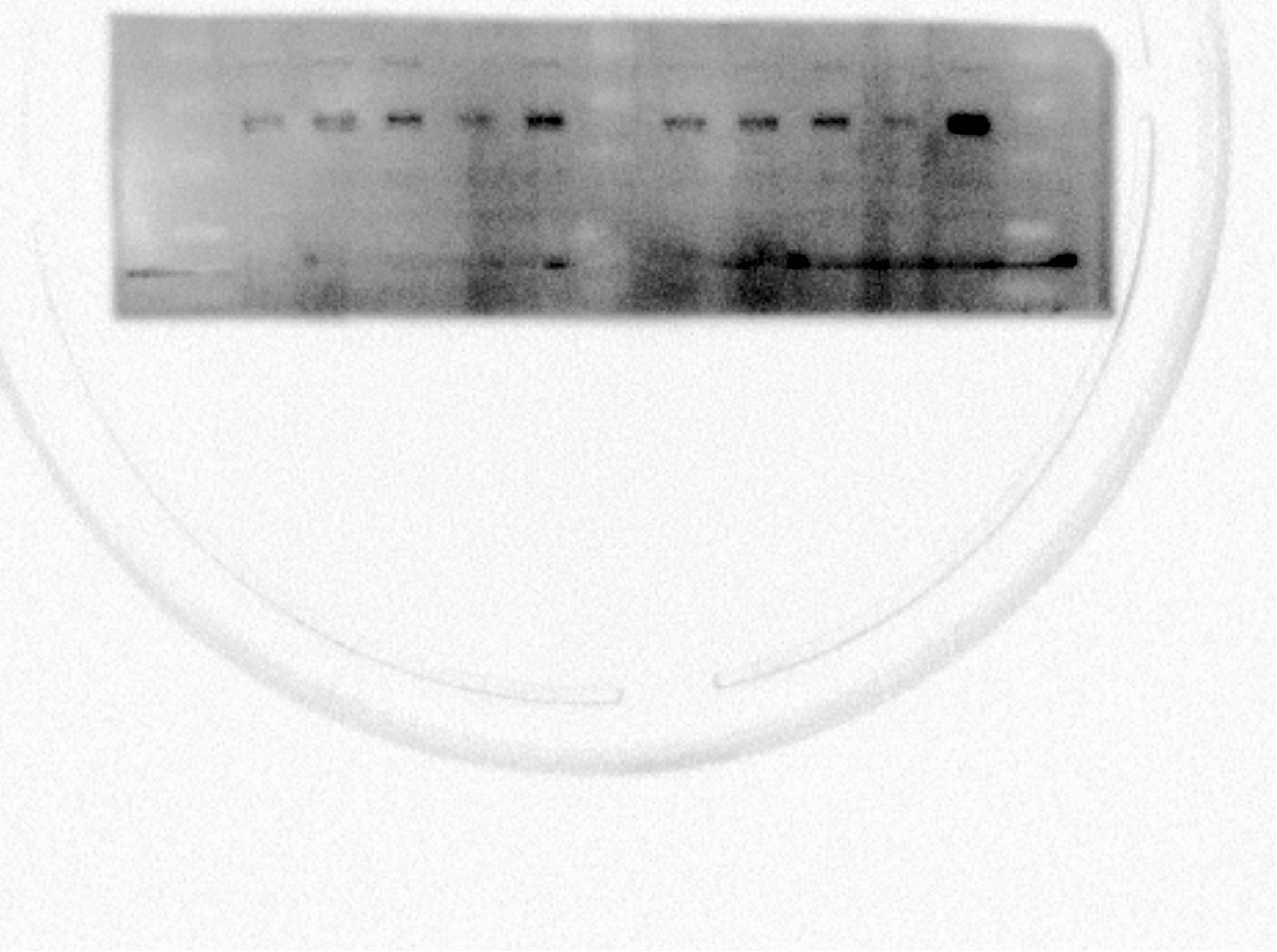
(e)
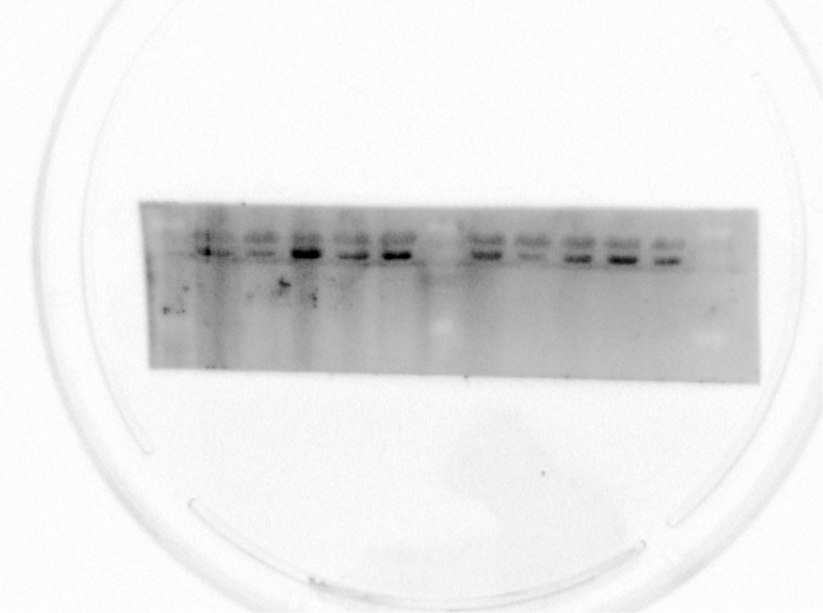


(3) β-actin

(a)
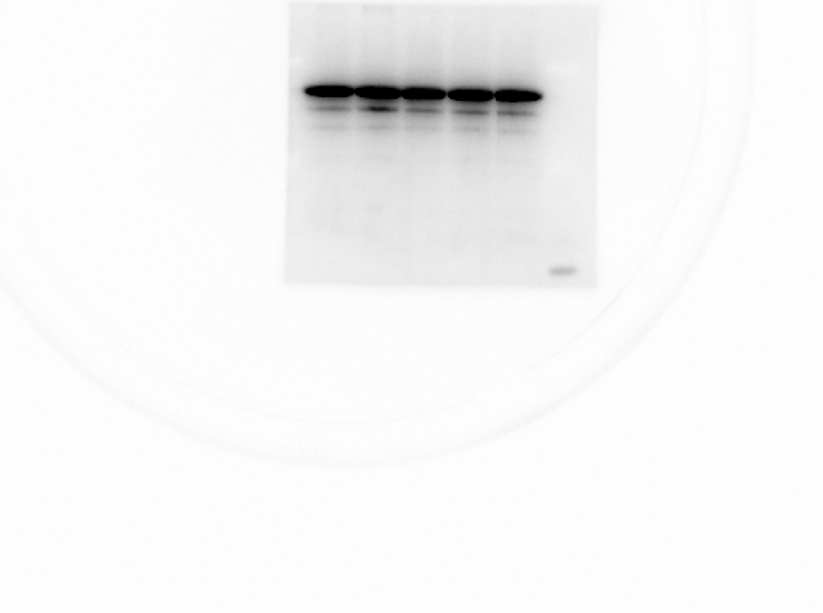
(b)
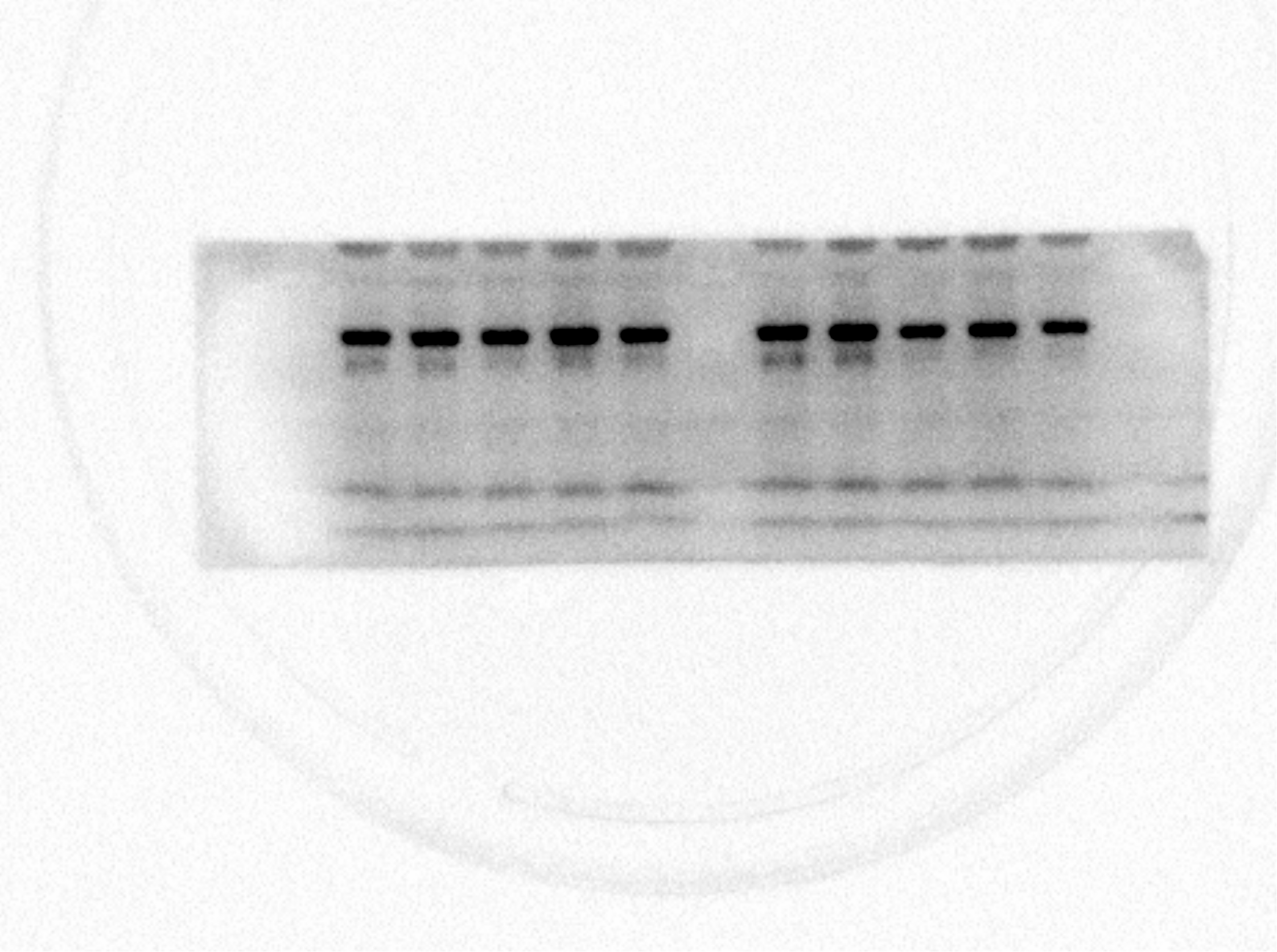
(c)
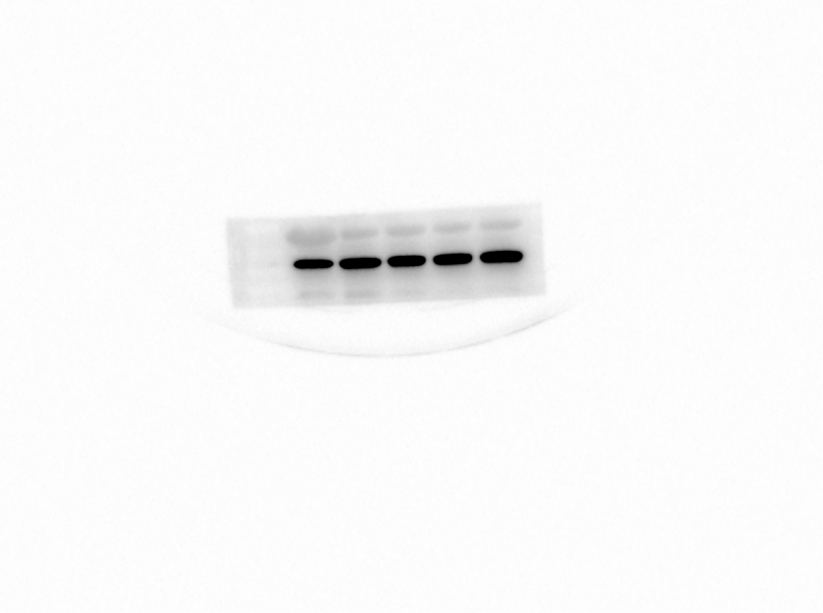


(d)
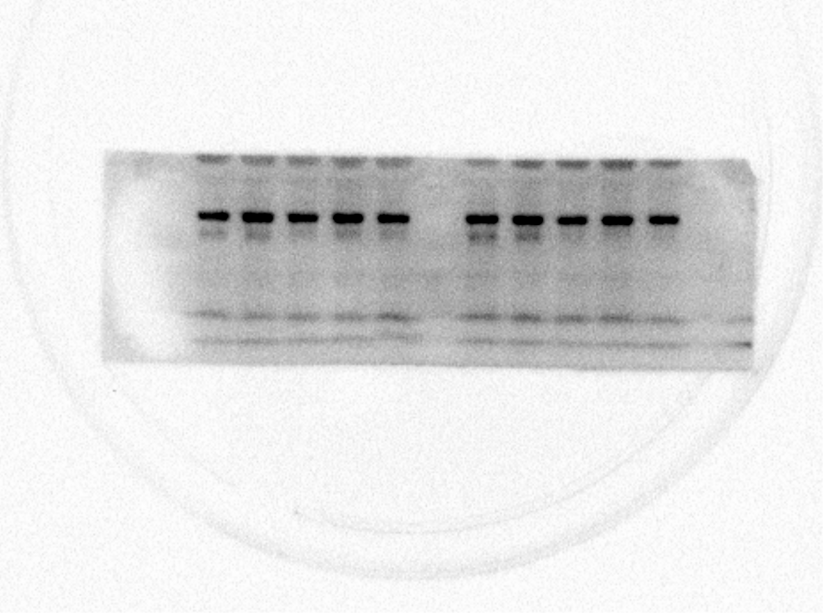
(e)
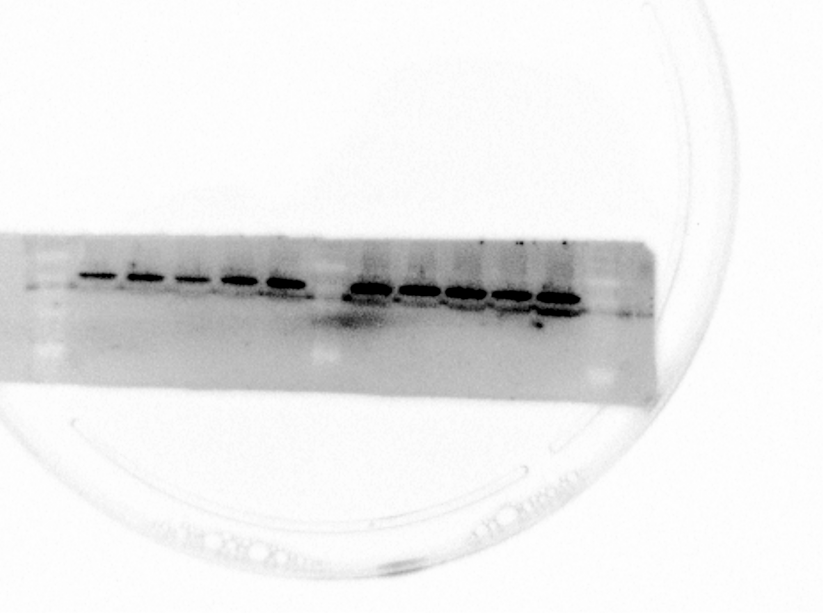


(4) Nrf2

(a)
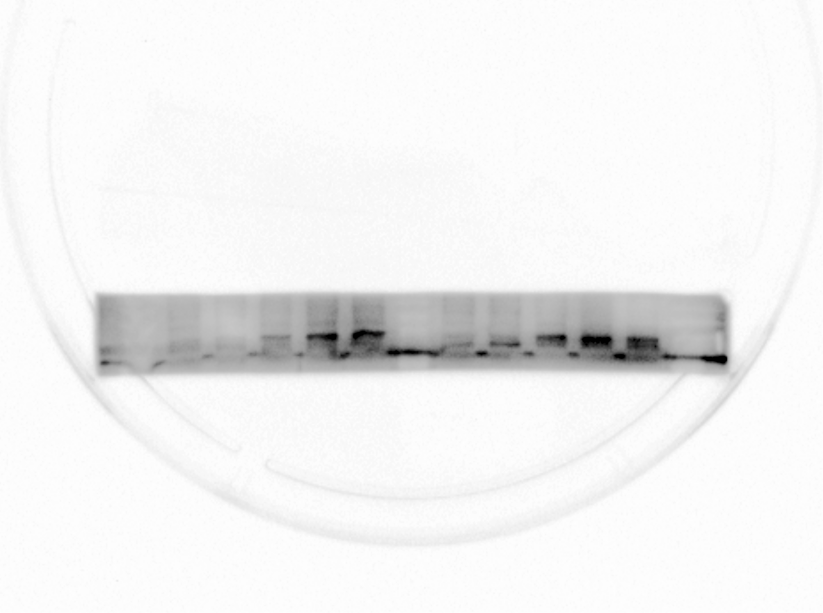
(b)
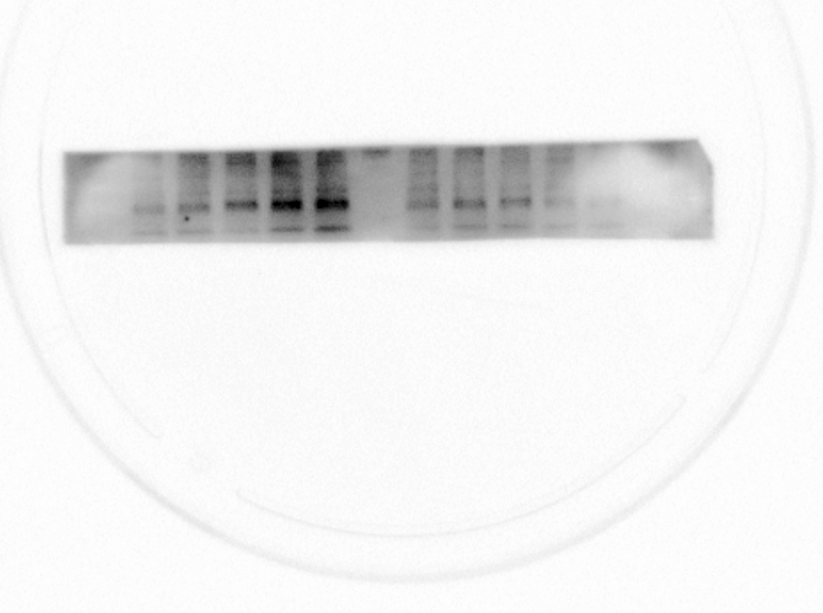


(c)
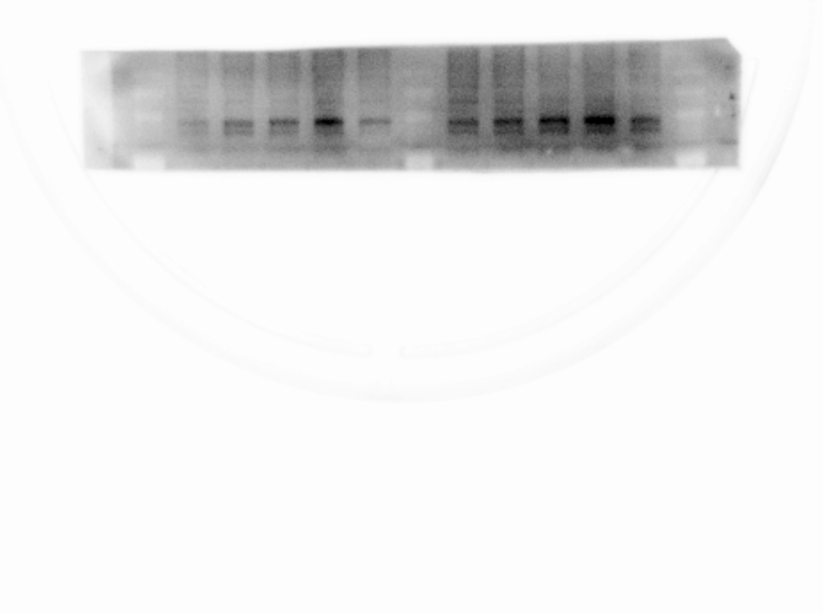
(d)
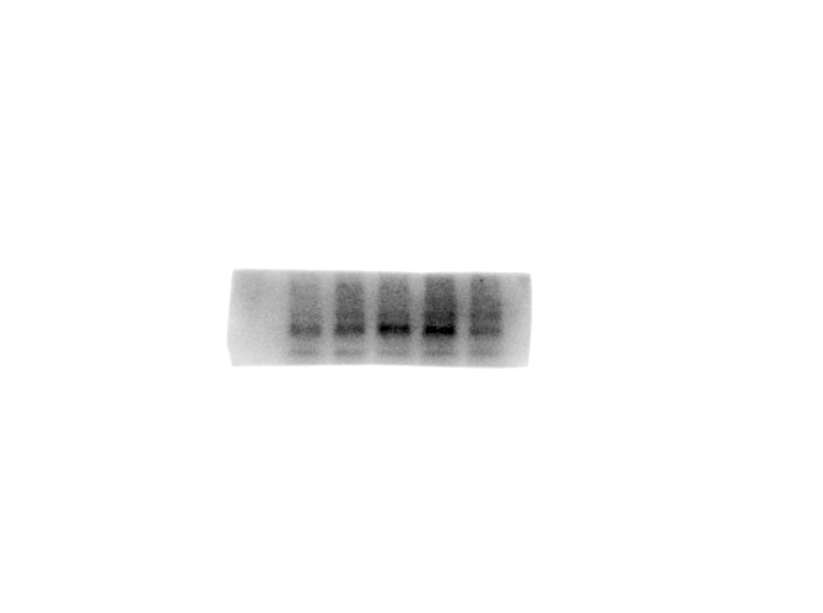


(4)β-actin

(a)
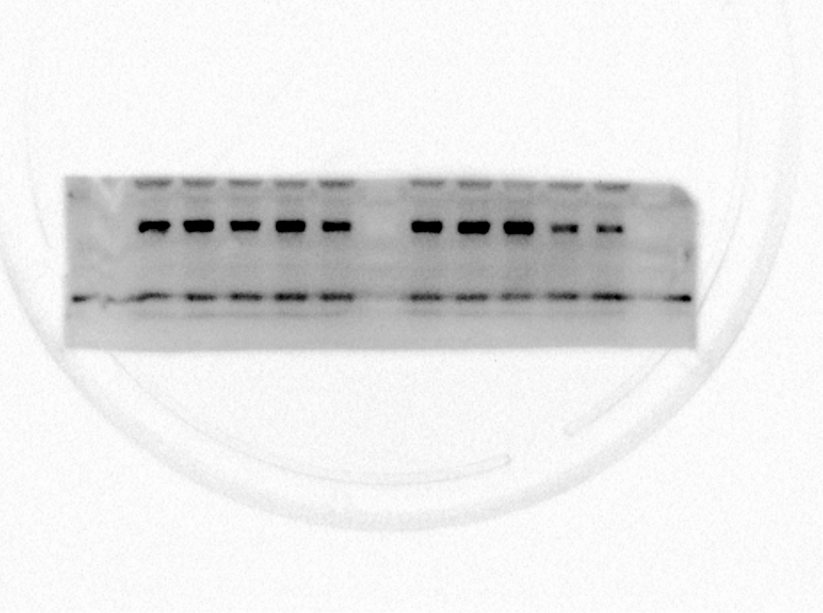
(b)
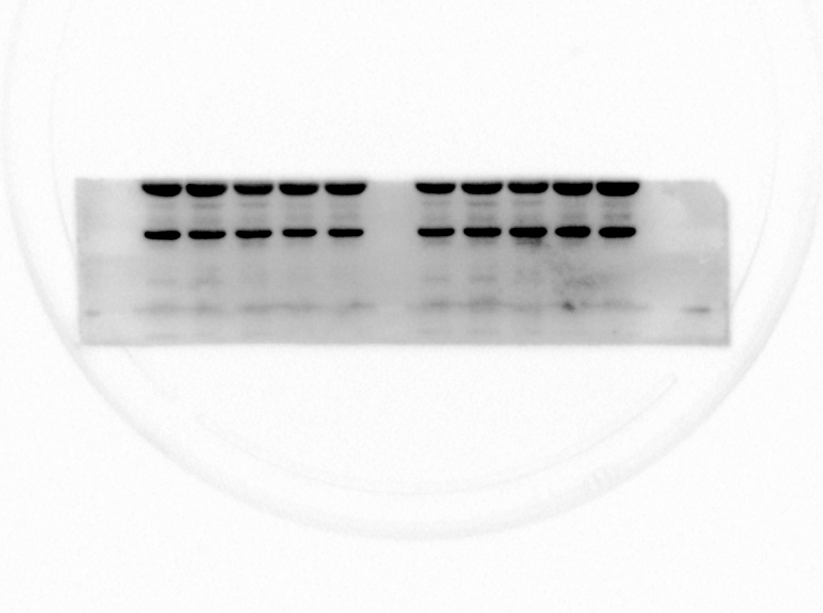


(c)
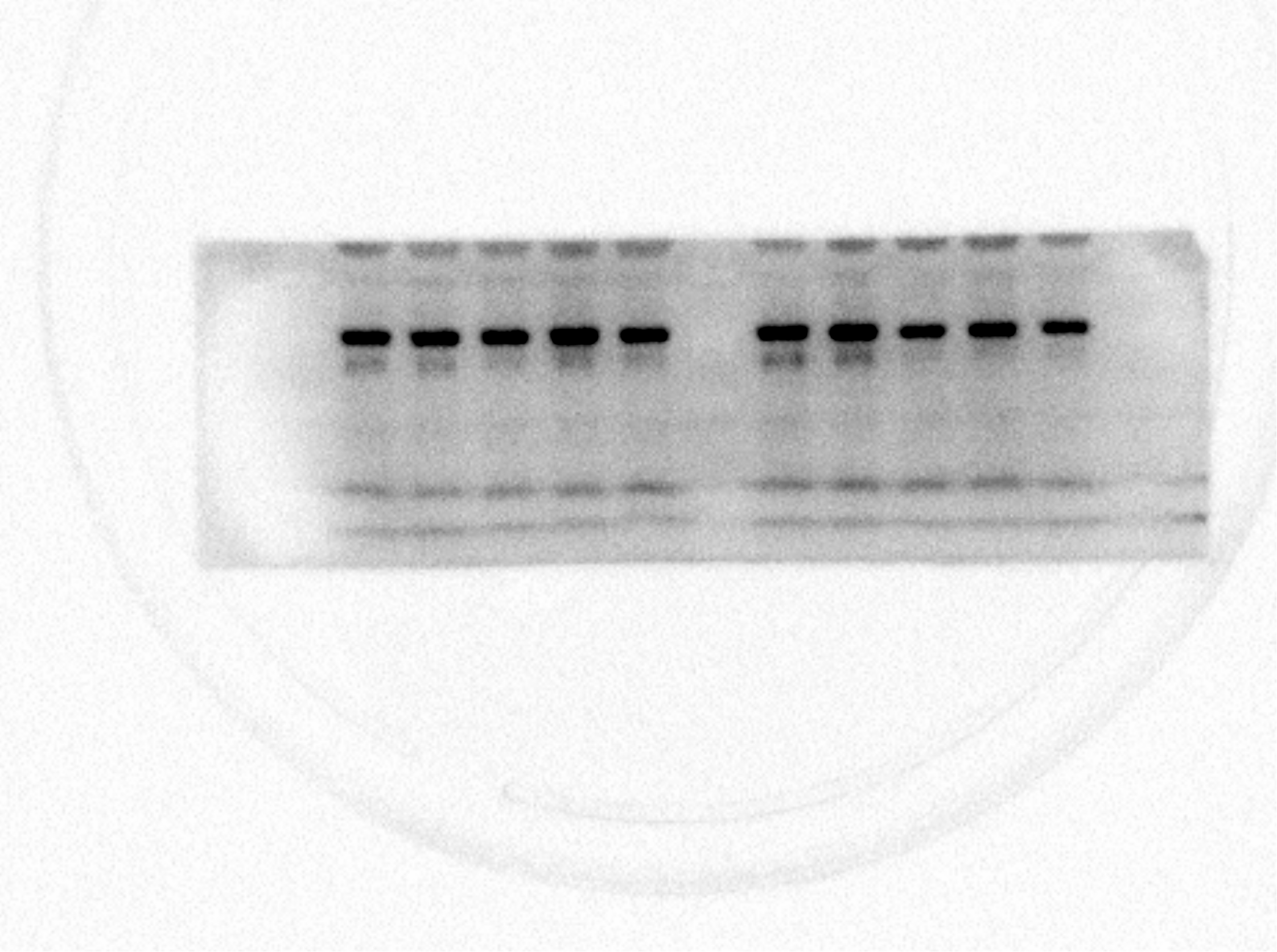
(d)
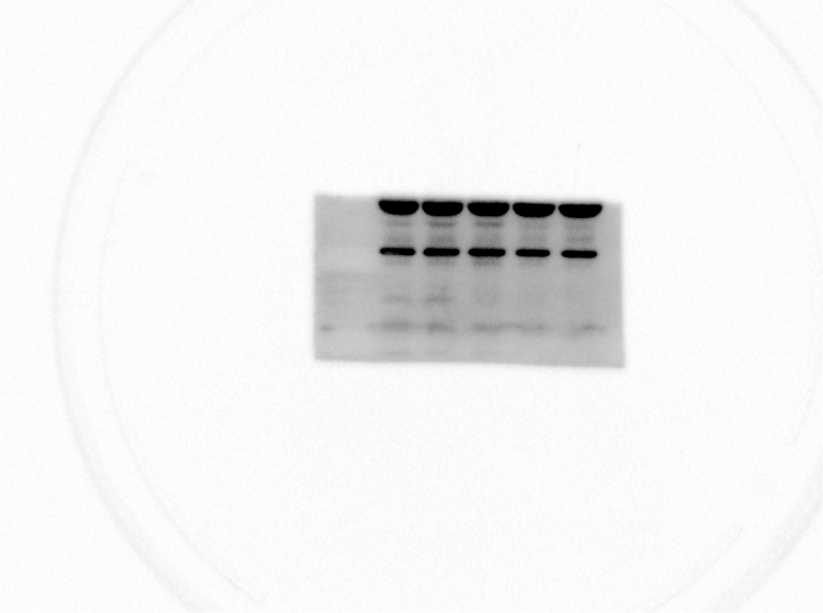


(5) HO-1

(a)
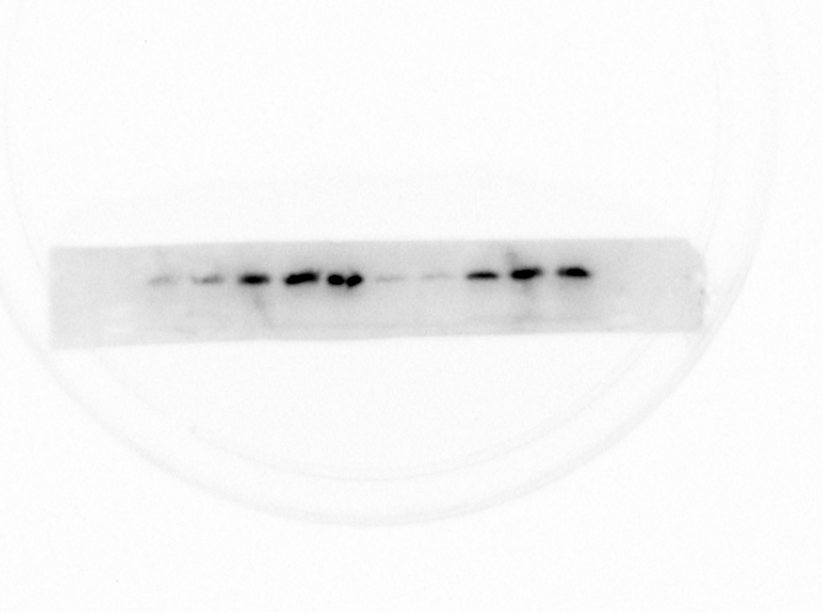
 (b)
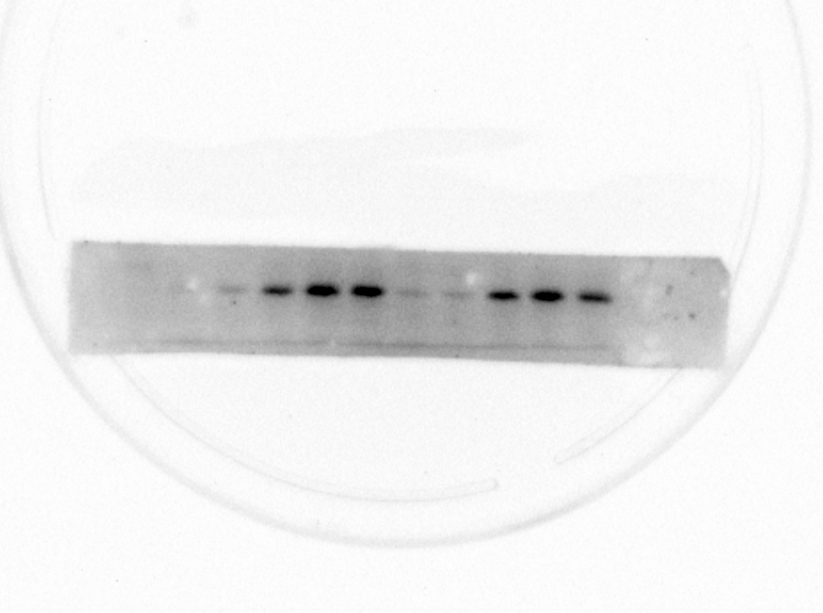


(c)
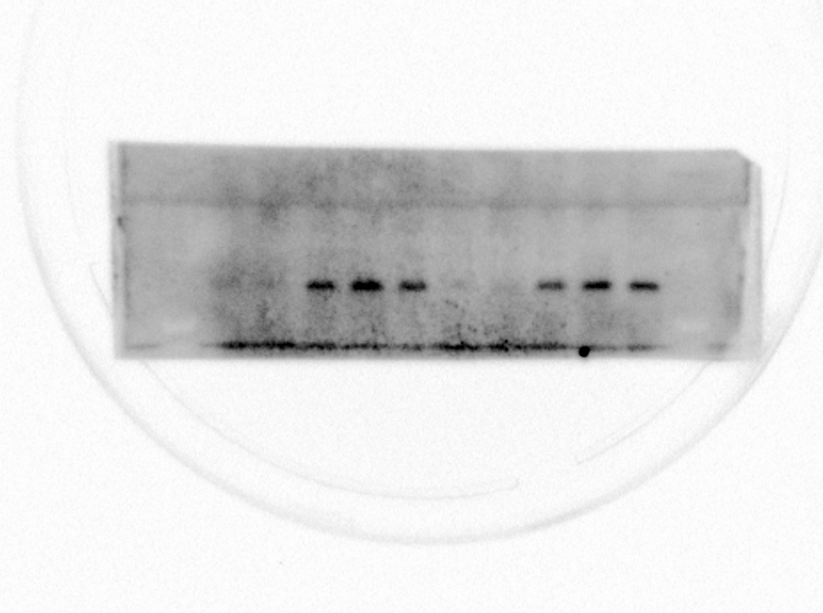
(d)
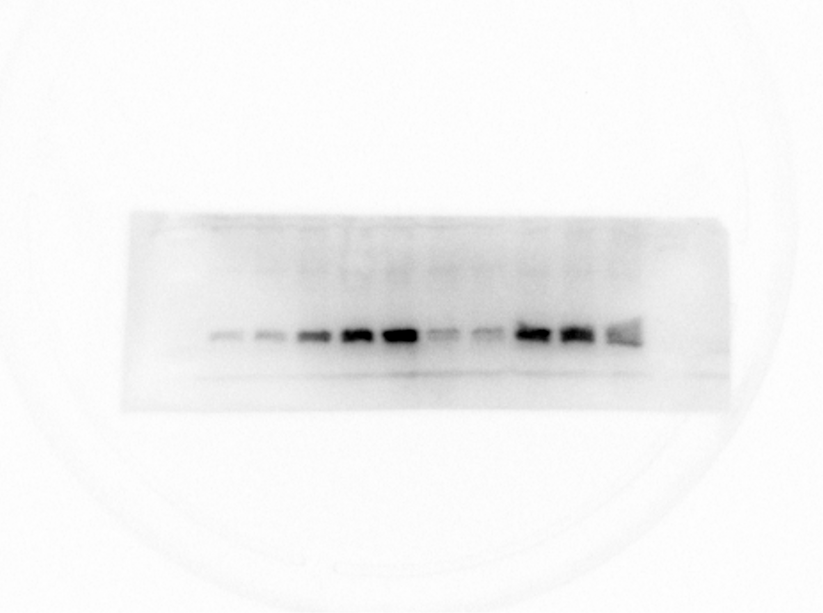


(5) β-actin

(a)
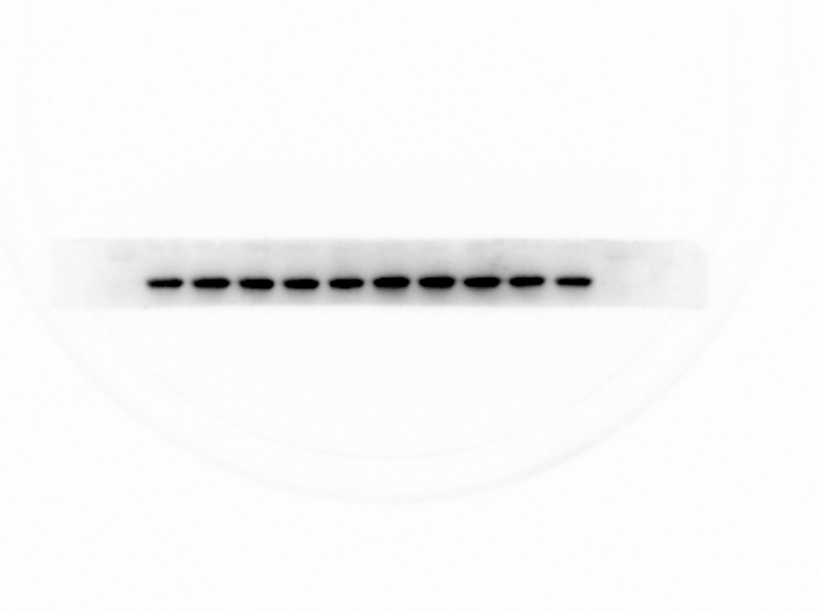
 (b)
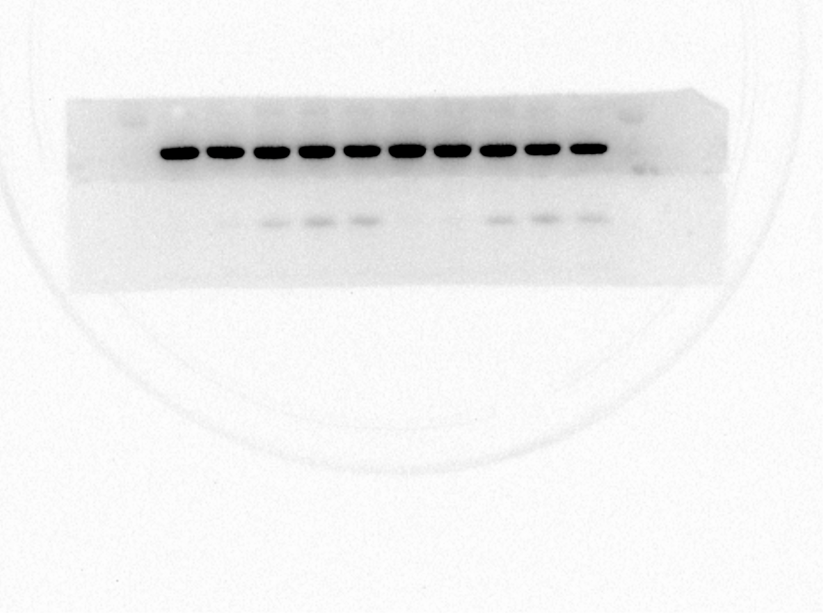


(c)
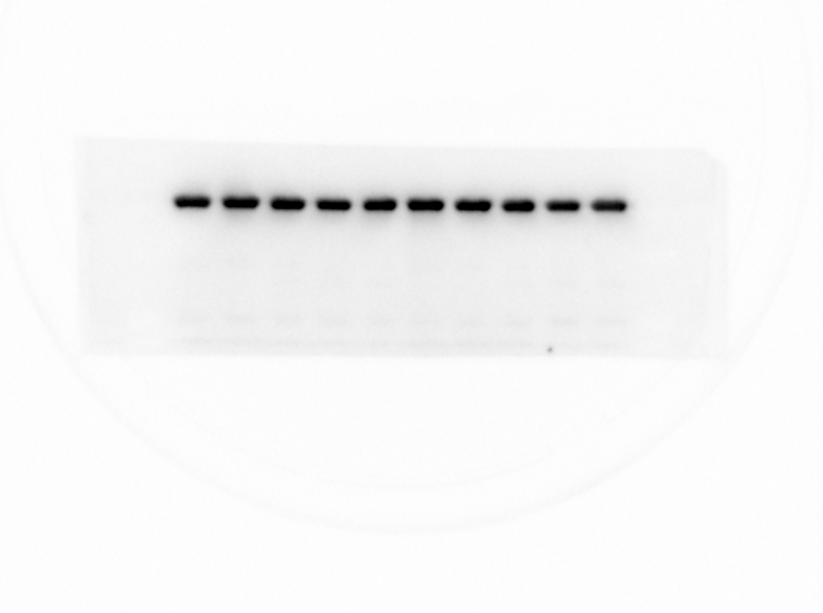
(d)
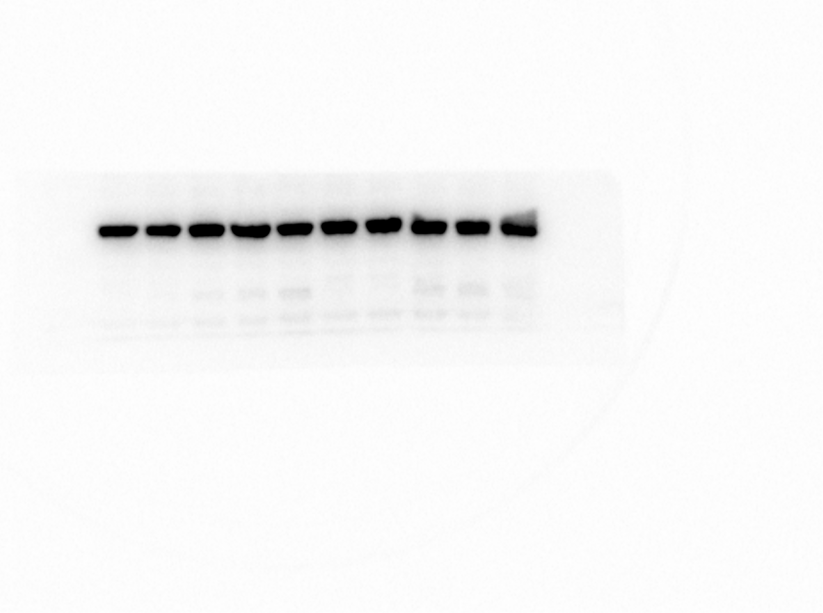

Supplement: Supplementary file 1 — Supplementary Material 1 (DOCX 32.2 MB) [file 10753_2025_2370_MOESM1_ESM.docx]
